# Lithospheric conductors reveal source regions of convergent margin mineral systems

**DOI:** 10.1038/s41598-022-11921-2

**Published:** 2022-05-17

**Authors:** Alison Kirkby, Karol Czarnota, David L. Huston, David C. Champion, Michael P. Doublier, Paul A. Bedrosian, Jingming Duan, Graham Heinson

**Affiliations:** 1 Mineral Systems Branch, Geoscience Australia, GPO Box 378, Canberra, 2601 Australia; 2 U.S. Geological Survey, Geology, Geophysics & Geochemistry Science Center, Denver, CO 80225 USA; 3 Department of Earth Sciences, University of Adelaide, Adelaide, SA 5005 Australia; 4 Present Address: GNS Science, Wairakei Research Centre, 114 Karetoto Road, Taupo, 3384 New Zealand; 5 Research School of Earth Sciences, Australian National University, Canberra, Australia

**Keywords:** Geochemistry, Geology, Geophysics

## Abstract

The clean energy transition will require a vast increase in metal supply, yet new mineral deposit discoveries are declining, due in part to challenges associated with exploring under sedimentary and volcanic cover. Recently, several case studies have demonstrated links between lithospheric electrical conductors imaged using magnetotelluric (MT) data and mineral deposits, notably Iron Oxide Copper Gold (IOCG). Adoption of MT methods for exploration is therefore growing but the general applicability and relationship with many other deposit types remains untested. Here, we compile a global inventory of MT resistivity models from Australia, North and South America, and China and undertake the first quantitative assessment of the spatial association between conductors and three mineral deposit types commonly formed in convergent margin settings. We find that deposits formed early in an orogenic cycle such as volcanic hosted massive sulfide (VHMS) and copper porphyry deposits show weak to moderate correlations with conductors in the upper mantle. In contrast, deposits formed later in an orogenic cycle, such as orogenic gold, show strong correlations with mid-crustal conductors. These variations in resistivity response likely reflect mineralogical differences in the metal source regions of these mineral systems and suggest a metamorphic-fluid source for orogenic gold is significant. Our results indicate the resistivity structure of mineralized convergent margins strongly reflects late-stage processes and can be preserved for hundreds of millions of years. Discerning use of MT is therefore a powerful tool for mineral exploration.

## Introduction

Growing global metal demand, coinciding with a decrease in new major discoveries of mineral deposits, has led to increasing efforts to improve the effectiveness of mineral exploration through the development of new techniques, datasets, and insights into deposit formation^1–3^. Case studies over the last decade have shown the MT method has promise as a means of narrowing the search space for some deposits formed in convergent margin settings, even in regions now covered by sedimentary or volcanic cover^4–9^. This advance is significant as deposits in modern and ancient convergent margins account for > 71% of all known copper, ~ 23% lead, ~ 39% zinc, ~ 75% of gold and are a source of critical minerals such as antimony, tungsten, bismuth, indium, molybdenum, rhenium, and tin^10,11^. Despite this importance systematic statistical studies of the MT method’s efficacy have not been attempted.

Convergent margins are associated with a characteristic suite of mineral systems formed at different stages of progressive margin development including volcanic hosted massive sulfide (VHMS; back-arc extension), porphyry copper (volcanic arc and post-subduction extension), and orogenic gold (orogenesis)^12^. Porphyry copper and VHMS systems are well characterised^12–14^, however, genetic models for orogenic gold deposits, particularly sources of fluids, are debated, with potential sources including crustal metamorphic devolatilization, the mantle or magmas^15^. Given that mineral precipitation along the ascent pathway of ore source fluids (e.g., graphite^16^) can change the resistivity of the lithosphere, MT imaging can shed light on the genesis of mineral deposits and improve exploration success in frontier areas.

The potential of a particular region to host mineral deposits can be assessed by combining maps of the components required to form a mineral system; for example, fertile mantle source regions and major lithospheric structures to guide fluid flow into the upper crust^17^. In order to use geophysical datasets and models, it is necessary to understand how geophysical signatures relate to mineral system components. For example, the transition between thick and thin lithosphere, as defined by surface wave velocity models converted to temperature, is globally strongly correlated with, and possibly controls, the location of sediment-hosted base metal deposits^11^. In the aforementioned MT case studies mineral deposits have been associated spatially with conductors in the mid to upper crust^5^, lower crust^4^ and lower lithosphere^18^. Lithospheric conductors can sometimes be traced to known mineral deposits at the surface and have been inferred to represent conductive minerals deposited along trans-lithospheric fluid pathways during transport of ore bearing fluids, which are then preserved for millions of years^4,5^. To improve the use of resistivity models as an exploration tool, it is necessary to quantify how the conductors relate to surface mineralization. For example, at which depths in the resistivity models do conductors correspond best to surface mineral deposition, does this relationship vary between deposit types, and do such relationships hold globally?

The qualitative association between lithospheric conductors and mineral deposits in a few mineral camps has provided motivation for continent-scale MT array programs including the Australian Lithospheric Architecture Magnetotelluric Project (AusLAMP), the SinoProbe program in China and the EarthScope program in the USA. From these programs, a growing number of resistivity models are being produced, covering areas of several thousand square kilometres^8,19–26^. This large coverage provides the first opportunity to quantify the relationship between lithospheric conductivity and mineral deposition on a global scale. By using global datasets our aim is to overcome regional differences between mineral provinces and discover generic insights. We include a focus on southeastern Australia, an excellent natural laboratory of an ancient convergent margin, endowed with 25 Mt Cu and ~ 6000 t Au^27–31^ with significant deposits of all three convergent margin mineral systems and excellent MT data coverage. We also analyse a combined dataset from the rest of the world. Our aim is to interrogate correlations associated with these deposit types in the mineral systems context, and to use the results to elucidate processes that control fluid and melt fluxes through the lithosphere.

## Data

**Figure 1:**
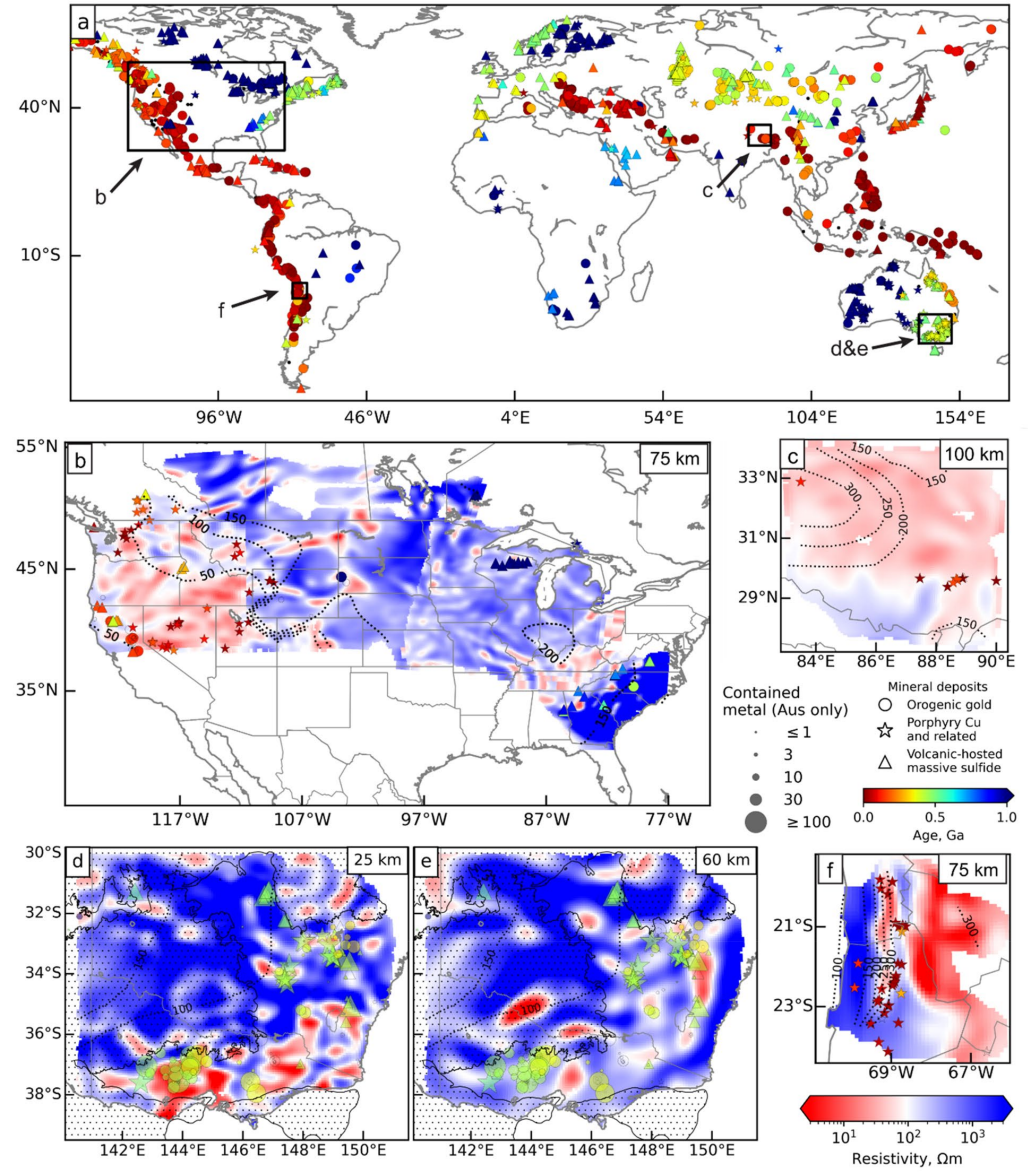
MT and deposit data used in this study. (**a**) Location of all resistivity models on a world map with deposits^11,27,31,34,35^ coloured by age; VHMS (triangles), porphyry copper (stars) and orogenic gold (circles). Other panels show resistivity models from (**b**) USA^19,21,22,26^ at 75 km depth, (**c**) China^33^ at 100 km depth, southeast Australia^20^ at (**d**) 25 km and (**e**) 60 km depth, and (**f**) South America^32^ at 75 km depth. Dotted lines represent lithosphere–asthenosphere boundary (LAB) depth (SL2013sv model)^11^ contours, labelled in km, shown on all resistivity models. In (**b**–**f**), the data are clipped to show resistivity, LAB and deposits onshore within a distance of 0.7° of a station, with deposits in Australia sized according to the maximum gold resource in tonnes or copper resource in kilotonnes. For d and e, extent of Mesozoic to Cenozoic sedimentary basin cover^36^ is shown by stipples. The maps in this figure were generated using the basemap modules (v1.2.2) within Matplotlib (v3.4.2)^37^ using MTPy^38,39^ to read the resistivity models.

For southeast Australia, we use the recently-published lithospheric resistivity model derived from AusLAMP data, which were collected on a 0.5° grid and can therefore resolve features down to ~ 50 km in scale^20^. We compile a total of 18 porphyry copper, 33 VHMS and 135 orogenic gold deposits with ≥ 1 t total Au resource or ≥ 1000 t total Cu (Methods). For our global analysis, we use published resistivity models covering areas containing, or prospective for, these same deposit styles^19,21,22,26,32,33^. We compile porphyry copper, VHMS, and orogenic gold deposit locations over these areas (146, 85 and 278 deposits respectively) and supplement these with the southeast Australian deposits (“Methods”)^11,27,31,34,35^. Visual inspection of the relationship between deposits and conductors reveals that there is a strong correspondence between lithospheric conductors and mineralisation. Figure 1 shows the key associations (see Supplementary Information for further details and additional maps). Whilst porphyry copper and VHMS deposits appear to correspond with upper mantle conductors, orogenic gold deposits appear to be strongly correlated with mid-crustal and asthenospheric conductors.

### Spatial analysis

**Figure 2:**
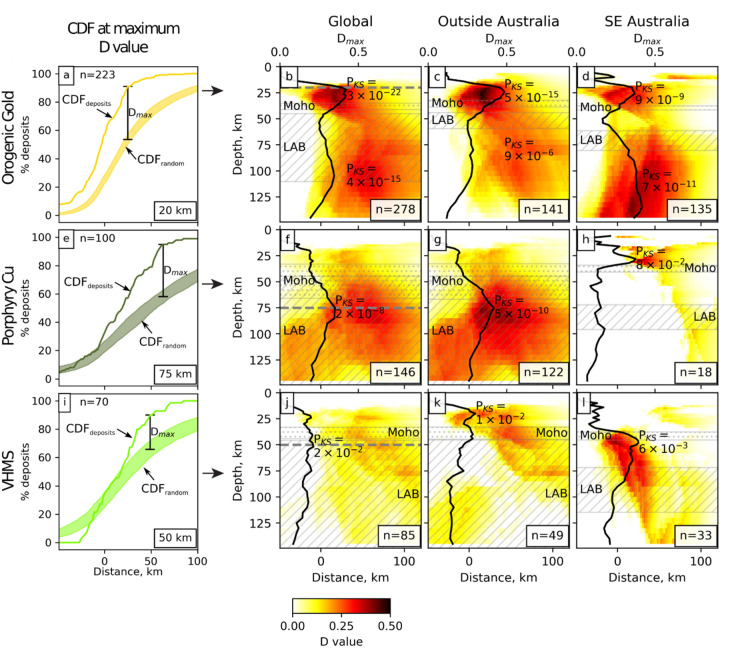
Cumulative distribution function of the distance of deposits (CDF_deposits_) compared to random locations (CDF_random_) from the 100 Ωm contour. (**a**–**d**) Orogenic gold, (**e**–**h**) porphyry copper, and (**i**–**l**) VHMS deposits. Left panel (**a**, **e**, **i**) shows CDF_deposits_, CDF_random_, with the maximum difference D_max_ = CDF_deposits_ − CDF_random_ indicated on the plots as a vertical bar, at a depth corresponding to the maximum D value in the global assessment of each deposit type (indicated by grey horizontal dashed lines in **b**, **f** and **j**). The range on the random locations represents the mean ± 1 standard error estimated from 100 realisations of random locations. Right three panels show the difference D = CDF_deposits_ − CDF_random_ in colour as a function of depth and distance of deposits from the contour, in resistivity models from the global dataset (**b**, **f**, **j**), outside Australia (**c**, **g**, **k**), and within southeast Australia only (**d**, **h**, **l**). In these images, D_max_ is shown as a function of depth as a black line, and the depths of the Moho^41–43^ and LAB^11,44^ are shown as grey semi-transparent dotted and hatched regions, respectively, with the range indicating the 10th to 90th percentile of depths directly beneath deposits. The n value indicates the number of deposits that were included in the analysis for that category, e.g. n = 278 in (**b**) indicates there are 278 orogenic gold deposits included in the global analysis.

We analyze the correlation between deposit locations and the resistivity models by computing a cumulative distribution function (CDF) of the distance of each deposit > 1t to the 100 Ωm contour at each depth in the resistivity models and comparing this to a CDF of distance to contour from random locations (see “Methods”). The 100 Ωm contour was used as it is the reference value for most of the compiled resistivity models—deviations from this value thus reflect data-driven changes to the resistivity models. Other resistivity contours and deposit size thresholds were tested with similar results (Supplementary section 3 and 4). The analysis was also repeated in southeast Australia on models with different vertical and horizontal smoothing, error floors and grid configurations, with similar results (Supplementary section 2). For example our analysis shows that 91% of orogenic gold deposits in the global dataset (n = 278 > 1 t) lie within 26 km of the 100 Ωm contour at 20 km depth (Fig. 2a). In comparison, the percentage that would be expected from random locations is 53 ± 4%. The difference, D = CDF_deposit_ – CDF_random,_ is shown in Fig. 2a,e,i, and the maximum value of D for any given depth (D_max_) allows us to quantitatively examine the correlation between deposit locations and modeled resistivity, as a function of depth and distance for each deposit type (Fig. 2b–d,f–h,j–l).

We use the two-sample Kolmogorov–Smirnov test^40^ to examine the statistical significance of the correlation (“Kolmogorov–Smirnov test”). It estimates that the probability, P_KS_, that the global distribution of orogenic gold deposits in relation to the resistivity contour at 20 km depth could be produced by chance, is 3 × 10^–22^, a local minimum (P_KS*min*_; by definition, a local maximum in D_max_). Another local minimum occurs at 95 km (P_KS*min*_ = 4 × 10^–15^), in the asthenospheric mantle. In the Australian dataset, orogenic gold deposits also show mid crust and convecting mantle peaks with similar P_KS*min*_ = 9 × 10^–9^ and 7 × 10^–11^ respectively, also indicating a strong correlation. For VHMS deposits, P_KS*min*_ occurs in the lithospheric mantle (~ 50 km depth) in both the Australian and global datasets with moderate values of P_KS*min*_ (6 × 10^–3^ and 0.02 respectively). Porphyry copper deposits, on the other hand, show a robust correlation globally, with P_KS*min*_ = 2 × 10^–8^ at 75 km depth, also within the lithospheric mantle, but a only mild correlation with conductors in Australia (P_KS*min*_ = 0.08).

### Causal mechanisms

Correlation between conductors at various depths in the lithosphere and deposits, and the difference in the nature of the correlation between deposit types, likely reflect differences in ore genesis.

Dry crustal and upper-mantle rocks typically have resistivities > 100 Ωm^45^. Metasomatism of the lower crust and upper mantle via introduction of fluids and melts is expected to occur proximal to deposits with a magmatic-hydrothermal fluid source and may reduce the resistivity in the ascent path^46^. Melt and free fluids are transient and disappear after the last tectonic event but metasomatism can persist over geologic time scales. Elevated water contents of commonly occurring minerals may explain some, but not all of the anomalies in the lower crust and upper mantle (Supplementary section 5). If interconnected (for example in fractures^47^ or on grain boundaries^48^), small volumes of a conductive material can substantially reduce rock resistivity. For example, 1–2% of an interconnected conductive material (≥ 10^4^ times more conductive than the host rock) can reduce resistivity of a rock by an order of magnitude or more^47,48^. Near mineral deposits, fractures and shear zones are likely to be abundant forming pathways for fluids through the lithosphere, increasing the interconnectivity of any conductive material present.

There is no equivocal universally agreed model for the fluid source for orogenic gold deposits, although exsolution of metamorphic fluids, mantle sources or magmatic fluids entering through trans-crustal fault zones appear to be most likely based on geochemical evidence^15^. Orogenic gold ore fluids are typically volatile-rich and have a reduced redox state (i.e. H_2_S is the dominant sulfur species), with typically 5–20 mol% CO_2_ and 0.01–0.36 mol% H_2_S^15,49,50^. In many cases the fluids are CH_4_-rich, indicating even more reduced fluids^51^. These reducing conditions favour the formation of conductive iron sulfide minerals such as pyrite (as low as 0.5 Ωm)^52^, and pyrrhotite (0.1–0.5 Ωm), and the high CO_2_ and/or CH_4_ content, together with the reduced state, could lead to the formation of graphite (< 1 Ωm)^53^. Thus, the resistivity anomalies associated with orogenic gold deposits are likely to be due to a combination of graphite and iron sulfides in the source region of these deposits^7^, which we argue based on resistivity models is in the middle to lower crust. The peak in spatial correlation between deposits and asthenospheric mantle conductors is unlikely to be genetically related to orogenic gold deposits as the deposits are millions of years older than the present-day thin lithosphere in these regions. In such regions the hot asthenospheric material at shallow levels is more conductive and mantle flow is likely to have contributed to uplift and exhumation of such deposits^54^ thereby explaining the observed correlation with conductors in the convecting mantle.

Globally, porphyry copper deposits are found in volcanic arc or post-orogenic extensional environments and are associated with metasomatism of the mantle by magmatic-hydrothermal fluids^55,56^. Some of the porphyry copper deposits considered in this study are in tectonically active environments (e.g. the U.S. Basin and Range extensional province; Fig. 1b), and thus the resistivity anomalies could be due to the presence of fluids and/or partial melt^57^. However, in other areas, an alternative explanation is required. In the source region in the upper mantle, it is likely that residual sulfide minerals such as pyrite and chalcopyrite (1–7 Ωm), and possibly pyrrhotite^58^ will be precipitated. These have been observed in varying amounts in metasomatised mantle xenoliths^59^. Furthermore, mantle metasomatism introduces hydrated minerals such as phlogopite (1 Ωm at ~ 900 °C)^60^ and amphibole (1–1000 Ωm at 550 °C)^61^; both minerals have been observed in xenolith samples from southeast Australia^62,63^. Thus, a strong association between lithospheric mantle conductors and porphyry copper deposits (in the global analysis) is unsurprising.

In contrast to global patterns, there is a weak correlation between conductors and porphyry copper deposits in southeast Australia, which are not only older (> 400 Ma compared to < 275 Ma outside Australia), but have both post-collisional (alkaline) and arc-related (calc-alkaline) types present. A possible explanation is that hydrous residue and residual sulfide present in the lower crust and upper mantle beneath deposits formed during arc magmatism is likely to be remobilized or translated during the formation of post-collisional porphyry copper deposits^64^, removing some of the resistivity signal in the ascent path.

**Figure 3:**
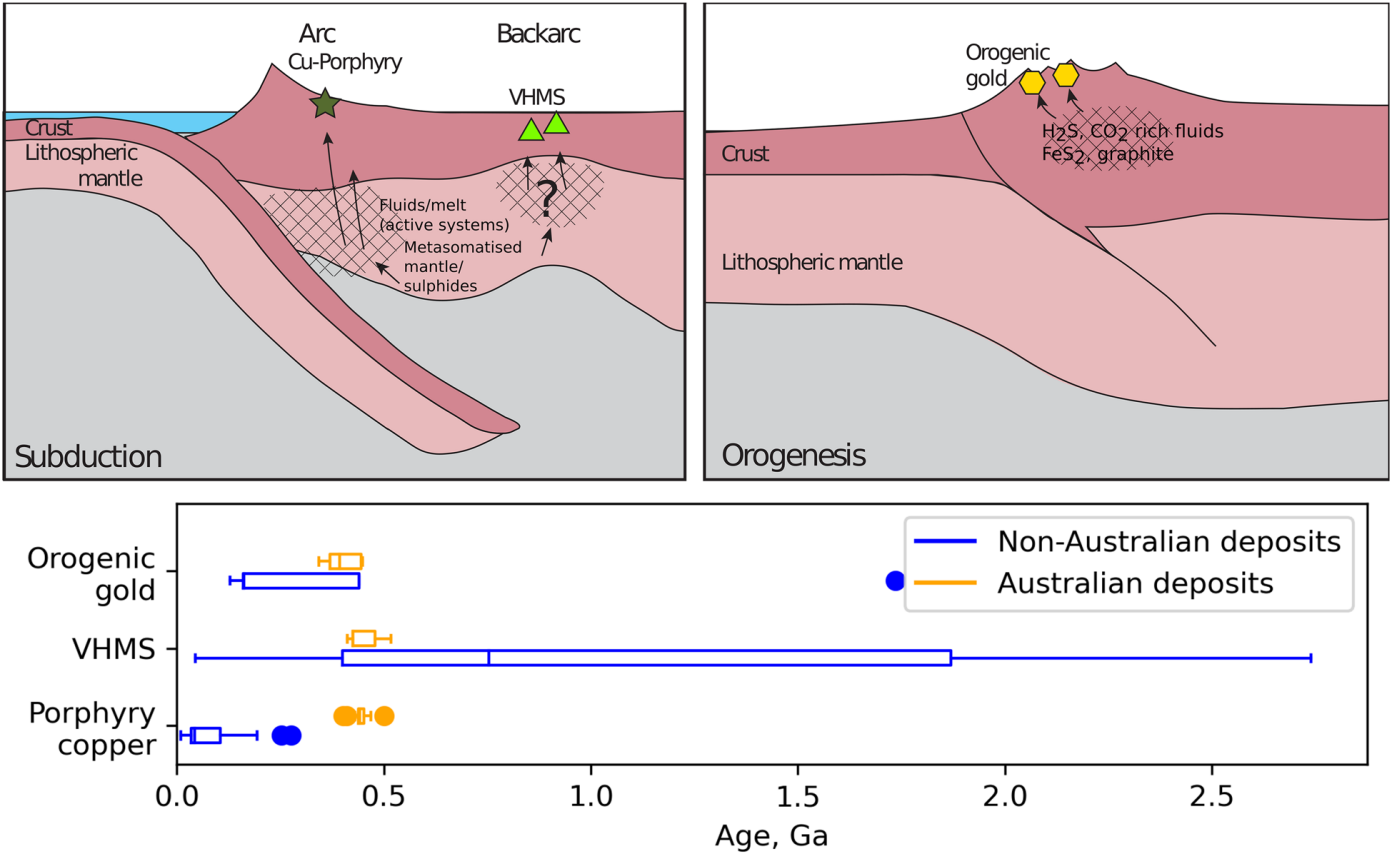
Top panel: schematic diagram showing the metal source regions beneath Porphyry Cu, VHMS, and Orogenic gold deposits. Porphyry Cu deposits are associated with arc environments, while VHMS deposits generally form in the backarc. Conductors likely represent metasomatism in the source regions of these deposits. Orogenic gold deposits are associated with carbonic, reduced fluids in orogenic environments, which our analysis suggests are sourced in the middle to lower crust. Bottom panel: age of the deposits analyzed, showing that, with the exception of the Australian deposits, the porphyry copper deposits are < 275 Ma while the VHMS deposits have a much wider range of ages. In the box and whisker plots, the boxes show first and third quartiles, the center line shows the median, the whiskers are limited to 1.5 time the inter quartile range from the median and filled dots show outliers.

VHMS deposits form in a back-arc environment from evolved seawater, with magmatic-hydrothermal fluids important in some mineral systems, which tend to be closer to the arc and rich in copper and gold^14^. Consistent with the shallow nature of these systems, the correlation pattern between conductors and VHMS deposits is less pronounced than for orogenic gold and porphyry copper. There is also a wide age range in these deposits; 0.04 to 2.74 Ga in the global dataset (Fig. 3); and in many regions they have been subjected to significant tectonism post deposition, for example the midcontinent VHMS deposits^65^, and those in the Appalachians^66^ and western Cordillera^67^ of the USA. Thus, the resistivity signal is less likely to be preserved compared to that for orogenic gold and porphyry copper deposits. However, the presence of a mild correlation peak between deposits and conductors in the lithospheric mantle may reflect hydrous melting and metasomatism of the mantle and lower crust during subduction, associated with back-arc basin formation. Such melting occurs under thinned lithospheric mantle, which then thickens during cooling, freezing in the mantle signatures. Alternatively, the mild correlation peak may simply reflect that mineral deposits in general, including VHMS, are often associated with crustal penetrating shear zones, which provide fluid pathways that can be preserved as conductive anomalies if the fluids contain and deposit conductive minerals.

## Reducing the search space

We demonstrate a strong correlation between mid- to lower crustal conductors and orogenic gold deposits and a moderate to mild correlation between upper-mantle conductors and porphyry copper and VHMS deposits, respectively. Further, we demonstrate that the locations of such conductors are geologically-feasible with respect to genetic models of ore deposit formation (Fig. 3). Thus, lithospheric mantle resistivity derived from continental-scale models may be used for first order targeting of porphyry copper deposits, and mid-crustal resistivity is useful in mapping fertile regions for orogenic gold deposits. For example, ~ 90% of orogenic gold and porphyry copper deposits occur within 26 and 58 km of the 100 Ωm contour at 20 and 75 km depth, respectively. The search space is reduced by nearly half, on this criterion alone. Follow up studies examining the links between conductors and orogenic gold deposits are warranted including more detailed MT studies which explore pathways between mid-crustal conductors and such deposits along with petrographic studies aimed at identifying conductive phases within the paragenetic sequence of deposit formation. Combining resistivity models with similar studies examining relationships in other datasets (e.g. passive seismic velocity models and potential field inversions) within a mineral systems context will further refine areas for exploration targeting.

## Conclusion

We show that there are robust correlations between orogenic gold and porphyry copper/copper–gold deposits and lithospheric electrical conductors imaged in magnetotelluric inversions. Orogenic gold mineralization, a late-stage convergent margin process, shows a very strong correlation with mid-crustal conductors. This is consistent with metals being sourced from devolatization of mid-crustal metamorphic rocks. Porphyry copper mineralization is associated with upper mantle conductors, likely reflecting the deep magmatic plumbing systems beneath these deposits. VHMS deposits show a weak association with lithospheric mantle conductors reaffirming the shallow hydrothermal circulations associated with these systems. Therefore, our results are geologically plausible and provide a framework for incorporating continent-scale resistivity models in mineral system targeting.

## Methods

### Resistivity model details

Table 1 details key information from the resistivity models included in this study. For more information, the readers are referred to the references in the table.

**Table 1:** Key details of resistivity models used in the statistical analysis presented in the paper.

Region	Continent	Reference	Nominal station spacing (km)	Inversion mesh resolution (km)	Inversion reference model (Ωm)	Mineral systems represented
Southeast Australia	Australia	^20^	55	7.5	100	Orogenic gold, VHMS, Porphyry copper
Central Plains	North America	^21^	70	10	100	Orogenic gold, VHMS, Porphyry copper
Mid-west	North America	^22^	70	10	100	Porphyry copper, VHMS
Pacific Northwest	North America	^19^	70	15	100	Orogenic gold, VHMS, Porphyry copper
Southeast US	North America	^26^	70	12	180	Orogenic gold, VHMS
Andes	South America	^32^	~ 30	10	100^a^	Porphyry copper
Tibetan Plateau	China	^33^	10–100	7.5	100	Porphyry copper

^a^100 Ωm halfspace with a 1000 Ωm predefined subducting slab.

### Deposit compilation

Our global deposit inventory builds on past compilations as follows. First, we take porphyry copper, VHMS, and orogenic gold deposits from Geoscience Australia’s Australian and global deposit database^27,35^. We then progressively supplement this, with the global compilation of porphyry copper and VHMS deposits used in analysis of deposit locations with respect to the LAB^11^, and a compilation of orogenic gold deposits from the USA (Supplementary Information), modified from an earlier compilation^34^.

We then supplement this database with deposits that include total contained resource data for New South Wales^28^, and historic production^29^ and estimated in-place resource^30^ from Victoria. We assign deposits to either orogenic gold, porphyry copper, or VHMS, other deposit styles are either minor in numbers (e.g. intrusion-related gold) or are associated with a range of primary sources (e.g. gold placer). The resource data from occurrences or deposits that are either within 0.01° of latitude or longitude of each other are combined. Small resource estimates with total gold resource < 1 t and copper resource < 1000 t (i.e. mineral occurrences) are then excluded. To avoid duplication, deposits are not added from successive sources if a deposit of the same type with the same location (to a precision of 0.01ׄ° of latitude or longitude) is already present in the database. Deposits are also not added if there is a deposit of the same type, with a matching name and within 1° of latitude/longitude, existing in the main database.

### Spatial analysis methodology

We analyze the correlation between deposit locations and the resistivity model, using a similar methodology applied to assess correlations between deposits and the depth of the lithosphere asthenosphere boundary (LAB)^11^. The resistivity models are first combined by interpolating onto a single grid. We then calculate the shortest horizontal distance from each deposit to the 100 Ωm contour at each depth in the resistivity model, where distance is defined to be negative for deposits located within the contour (i.e. lower resistivity) and positive outside. Other values were also trialed with similar results (Supplementary section 2). We then calculate a cumulative distribution function (CDF) for each depth in the model, and for each deposit classification (CDF_deposit_; Fig. 2 of main manuscript). We compare these to the CDF for distances calculated from an equivalent number of random locations (mean of 100 realizations) filtered to be onshore and, in Australia, outside areas of Mesozoic to Cenozoic sedimentary basin cover (CDF_random_).

### Kolmogorov–Smirnov test

The two-sample Kolmogorov–Smirnov (K-S) test^40^ is used to examine whether the difference between two populations is significant, given their respective sizes. The K-S test was chosen as it is a simple non-parametric measure, where the null hypothesis distribution (i.e. that Cumulative Distribution Functions (CDFs) are drawn from the same distribution) is usefully approximated for non-zero values and it is valid for small sample sizes of deposit locations considered in this study^68^. First a D value, the maximum difference between two CDFs, expressed as a proportion in the range 0 to 1 is calculated. The K-S test estimates the probability that a given D value could accidentally occur if the two CDFs had been drawn from the same population. The probability P_KS_ is approximated by the following equation:

$${P}_{KS}\approx \mathrm{exp}\left(\frac{-2pq{D}^{2}}{p+q}\right)$$

where p and q are the number of samples in each CDF. For each K-S test, we seed 100 populations of random locations with the same number of samples and compute a CDF for each of these populations as well as the true deposit locations. A moderate to high value (e.g. > 0.05^69^) indicates there is a reasonable chance that the distribution of distances from the contour could be drawn from a population of random locations and thus the correlation between deposits and conductors is weak. Conversely, very low probabilities indicate it is highly unlikely the distribution of distances from the contour could be pulled from random and thus the correlation is strong. The D value used in the calculations is the maximum difference between the CDF for the true deposit locations and the mean CDF for the 100 random locations.

## Supplementary Information

Supplementary Information 1.

Supplementary Information 2.

## Data Availability

The MT data that were used to generate all four of the US MT models are available at the IRIS Data Management Center: http://ds.iris.edu/spud/emtf/. Resistivity model of central USA^21^ is available from http://ds.iris.edu/ds/products/emc-tho-mt-2021/. The three other USA resistivity models^19,22,26^ are available by request through the authors or through the citations provided in the manuscript. The MT data used to generate the Southeast Australia model^20^ are available from 10.11636/Record.2020.011 and 10.11636/Record.2018.021. The resistivity model of southeast Australia^70^ is available from 10.26186/131889. The resistivity models over parts of China and South America^32,33^ can be accessed from the citations provided in the manuscript. The merged deposit datasets used for the analysis are provided as Supplementary data. Geoscience Australia’s global and Australian deposit databases^27,35^ can be accessed through Geoscience Australia’s eCat repository at 10.11636/Record.2022.010 and 10.11636/Record.2021.020. The source for the other deposit data can be found with the citations provided in the manuscript^11,27–29,31,34^.

## Code availability

The code used to perform the analysis presented in the manuscript can be accessed through Geoscience Australia’s GitHub repository here https://github.com/GeoscienceAustralia/mineral-stats-toolkit.
