# Supplementary material for: Lithospheric conductors reveal source regions of convergent margin mineral systems
Source: Sci Rep. 2022 May 17;12:8190. doi: 10.1038/s41598-022-11921-2 (PMC9114405; doi:10.1038/s41598-022-11921-2)
Supplement: Supplementary file 1 — Supplementary Information 1. [file 41598_2022_11921_MOESM1_ESM.docx]

SUPPLEMENTARY MATERIAL

1. Additional depth slices


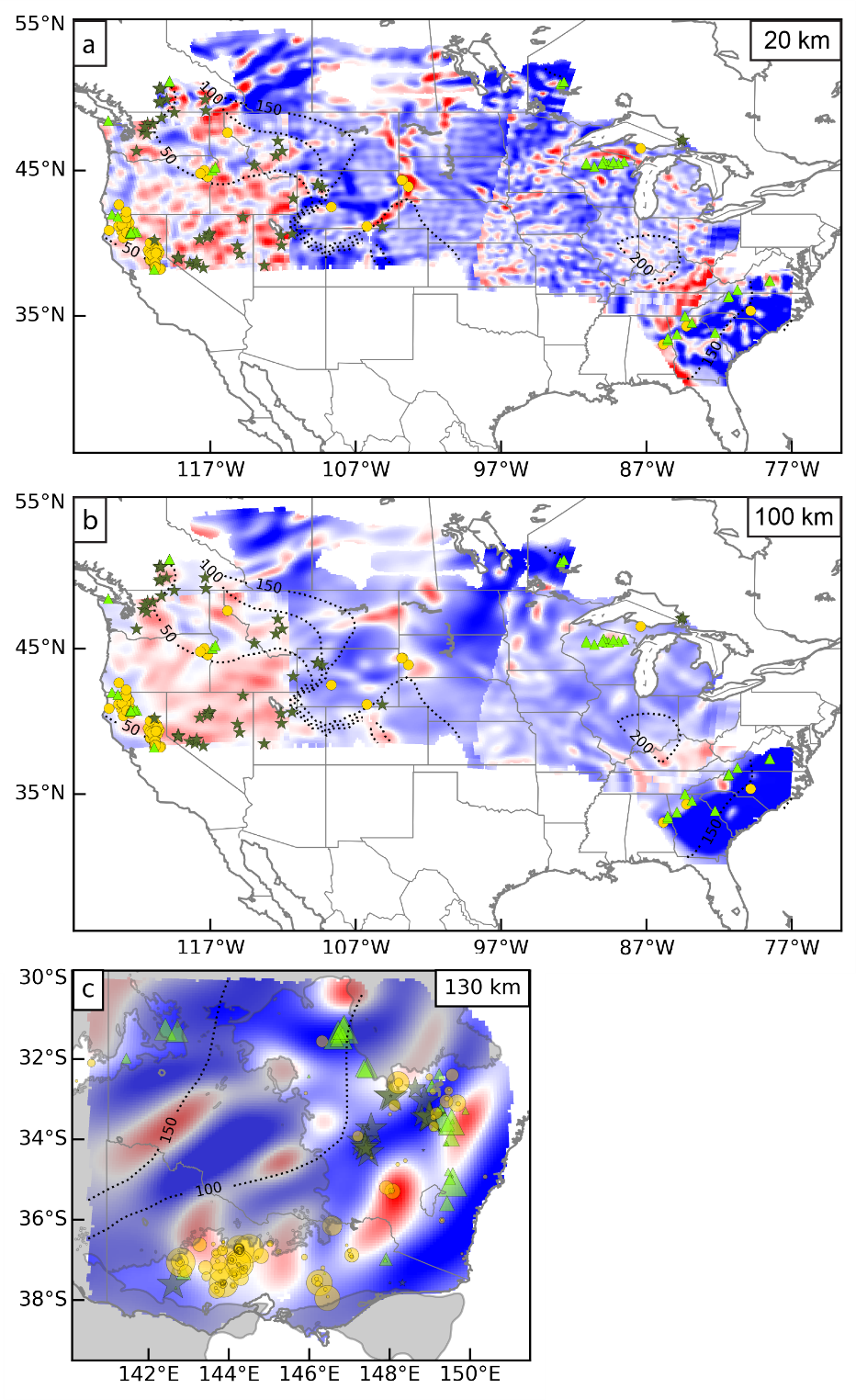


Figure S1: Additional resistivity model depth slices at (a) 20 km and (b) 100km (USA) and (c) 130 km (Australia). Symbology as for Figure 1 of main manuscript.

1. Analysis of different resistivity models in southeast Australia

In this section, we describe the results of statistical analysis carried out on resistivity models that were produced in southeast Australia to understand the sensitivity of the MT inversion to the input parameters. These models include runs with different error floors, different vertical grid configurations, different vertical and horizontal model covariance (smoothing parameter). We also ran four inversions each with 25 % of the stations removed at random (runs 165 to 168). We include here depth slices and cumulative distribution as a function of depth for each of these models. Table 1 details inversion parameters.

Table 1: Inversion parameters for sensitivity tests carried out in southeast Australia.

| **Model number** | **Number of stations** | **Z cellsize top** | **Z num constant cells** | **Z increase factor core** | **Horizontal covariance** | **Vertical covariance** | **Reference model Ωm** | **Error floor Z %** | **Error floor Tipper** | **RMS misfit** | **RMS misfit z** | **RMS misfit tip** |
| --- | --- | --- | --- | --- | --- | --- | --- | --- | --- | --- | --- | --- |
| 144 | 298 | 200 | 10 | 1.046 | 0.6 | 0.6 | 100 | 3 | 0.02 | 2.22 | 2.18 | 2.32 |
| 147 | 298 | 100 | 10 | 1.056 | 0.6 | 0.6 | 100 | 5 | 0.02 | 2.01 | 1.93 | 2.19 |
| 145 | 298 | 200 | 10 | 1.046 | 0.6 | 0.6 | 100 | 7 | 0.02 | 1.48 | 1.34 | 1.74 |
| 148 | 298 | 200 | 10 | 1.046 | 0.6 | 0.6 | 300 | 5 | 0.02 | 1.92 | 1.78 | 2.19 |
| 146 | 298 | 50 | 9 | 1.065 | 0.6 | 0.6 | 100 | 5 | 0.02 | 1.87 | 1.78 | 2.04 |
| 164 | 298 | 20 | 1 | 1.046 | 0.6 | 0.6 | 100 | 5 | 0.01 | 2.24 | 2 | 2.71 |
| 165 | 223 | 20 | 1 | 1.046 | 0.6 | 0.6 | 100 | 5 | 0.02 | 2.38 | 2.42 | 2.28 |
| 166 | 224 | 20 | 1 | 1.046 | 0.6 | 0.6 | 100 | 5 | 0.02 | 1.9 | 1.8 | 2.09 |
| 167 | 223 | 20 | 1 | 1.046 | 0.6 | 0.6 | 100 | 5 | 0.02 | 1.89 | 1.81 | 2.05 |
| 168 | 224 | 20 | 1 | 1.046 | 0.6 | 0.6 | 100 | 5 | 0.02 | 1.97 | 1.9 | 2.12 |
| 157 | 298 | 20 | 1 | 1.046 | 0.4 | 0.4 | 100 | 5 | 0.02 | 1.82 | 1.73 | 1.99 |
| 156 | 298 | 20 | 1 | 1.046 | 0.5 | 0.5 | 100 | 5 | 0.02 | 1.7 | 1.61 | 1.89 |
| 158 | 298 | 20 | 1 | 1.046 | 0.7 | 0.7 | 100 | 5 | 0.02 | 2.26 | 2.23 | 2.34 |
| 169 | 298 | 20 | 1 | 1.046 | 0.6 | 0.2 | 100 | 5 | 0.02 | 2.25 | 2.18 | 2.4 |
| 170 | 298 | 20 | 1 | 1.046 | 0.6 | 0.4 | 100 | 5 | 0.02 | 2 | 1.91 | 2.2 |
| 171 | 298 | 20 | 1 | 1.046 | 0.6 | 0.8 | 100 | 5 | 0.02 | 3.47 | 3.79 | 2.62 |

While there are minor differences in the correlation patterns between deposits and the different resistivity models, and differences in the strength of the correlation as measured by P_KS_*_min_*, the broad trends remain for the large majority of these, i.e. weak peak in the difference, D, between the cumulative distribution function of the distance from the 100 Ω.m contour from deposits (CDF_deposits_) compared to random locations (CDF_random_) in the mid to lower crust for porphyry copper, strong peaks in the mid-lower crust and asthenosphere for orogenic gold, and weak peak in the lithospheric mantle for VHMS.


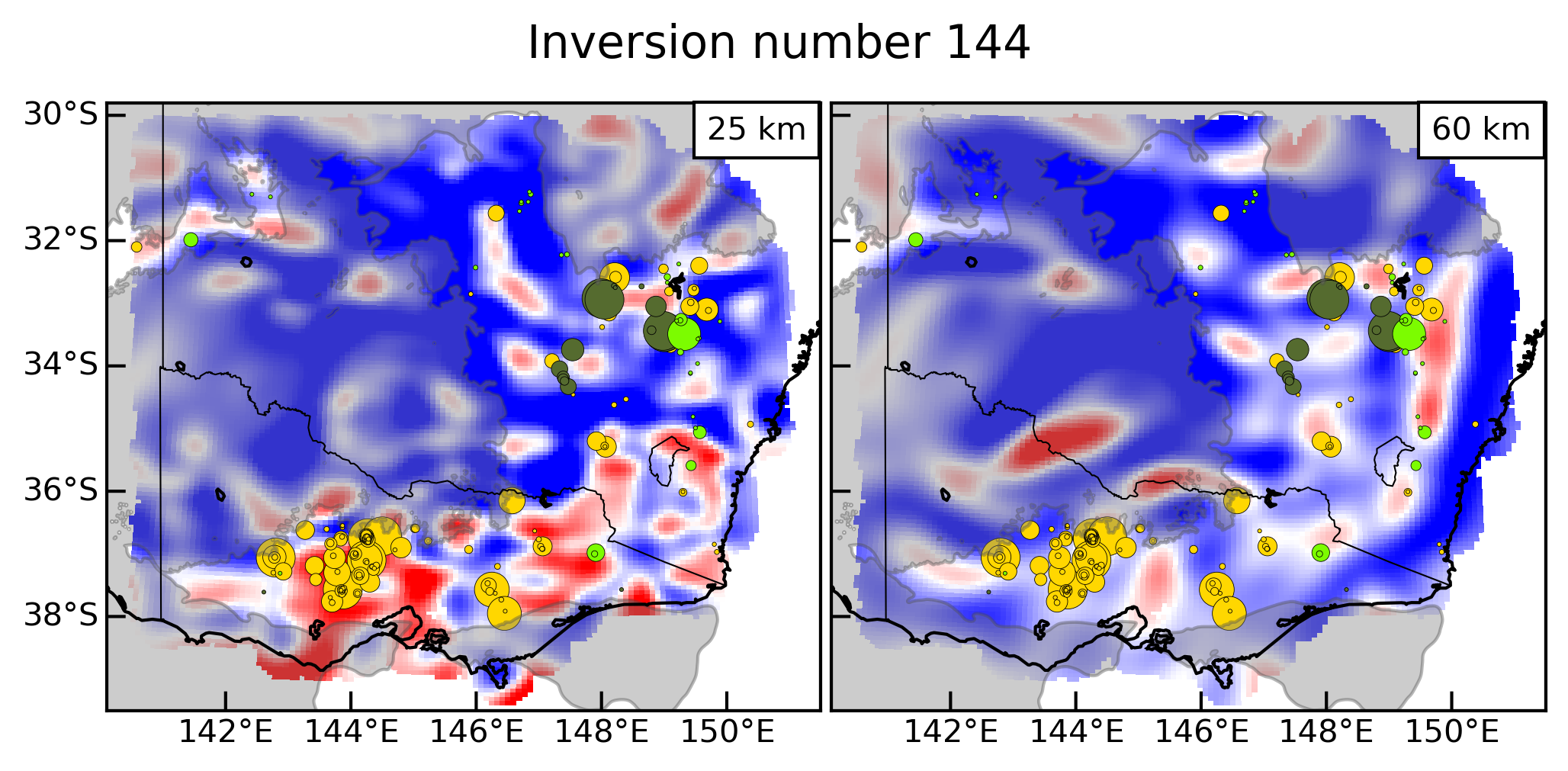

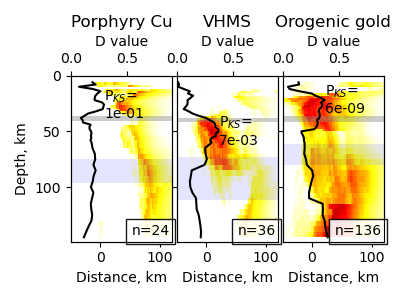


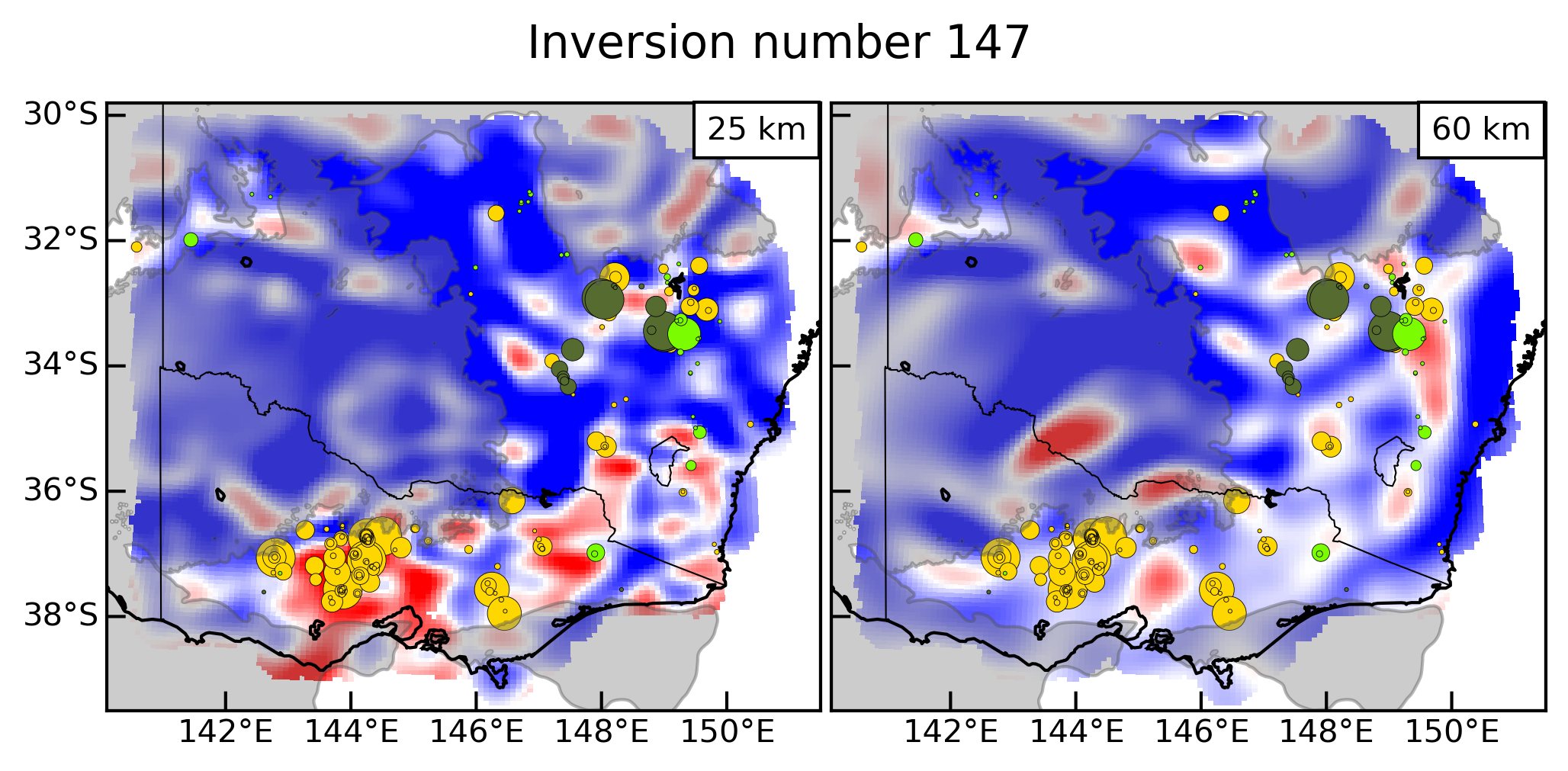

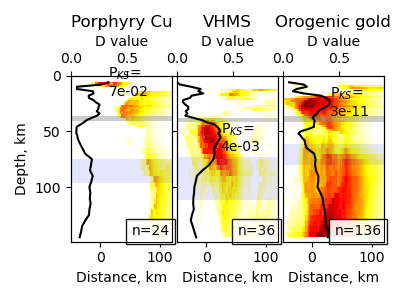


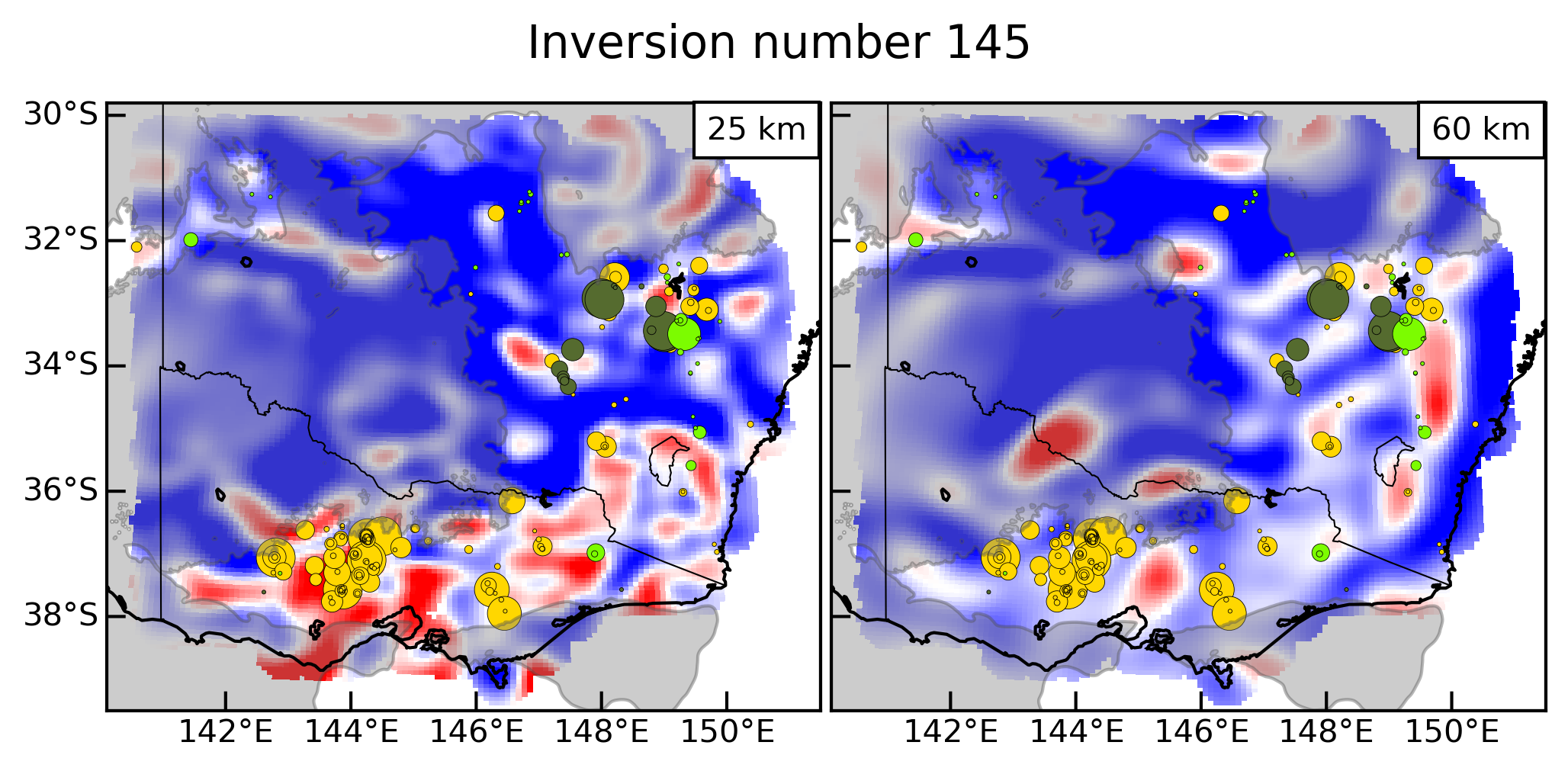

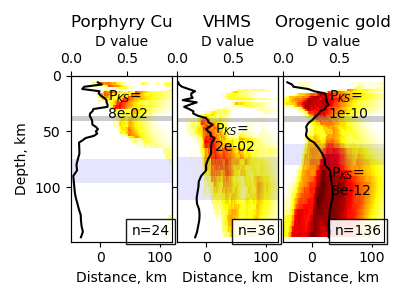


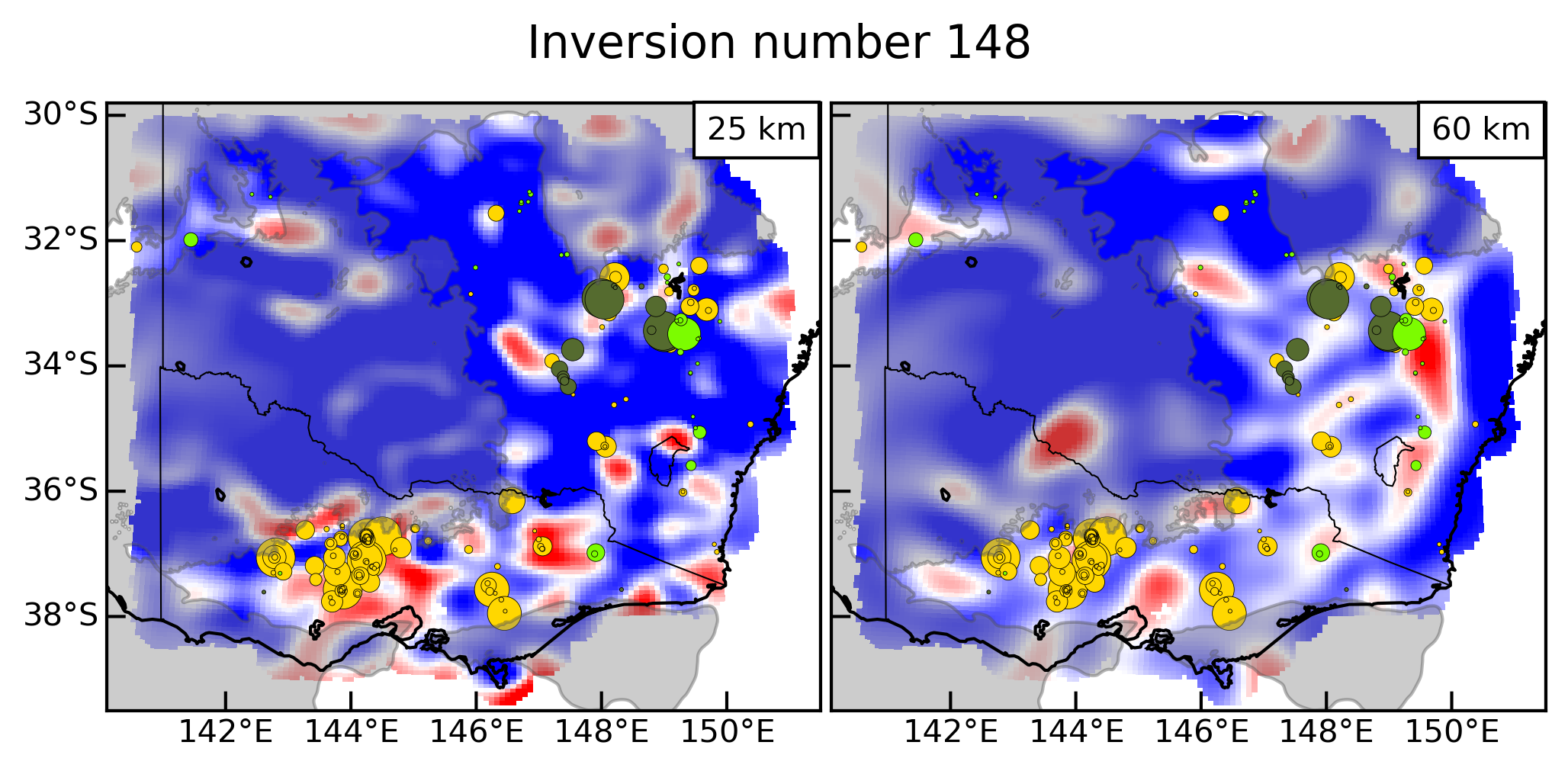

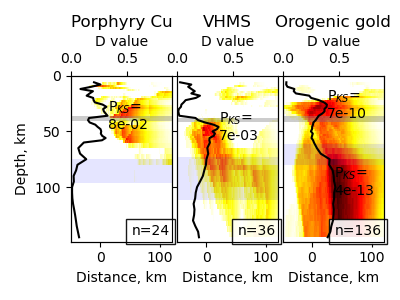


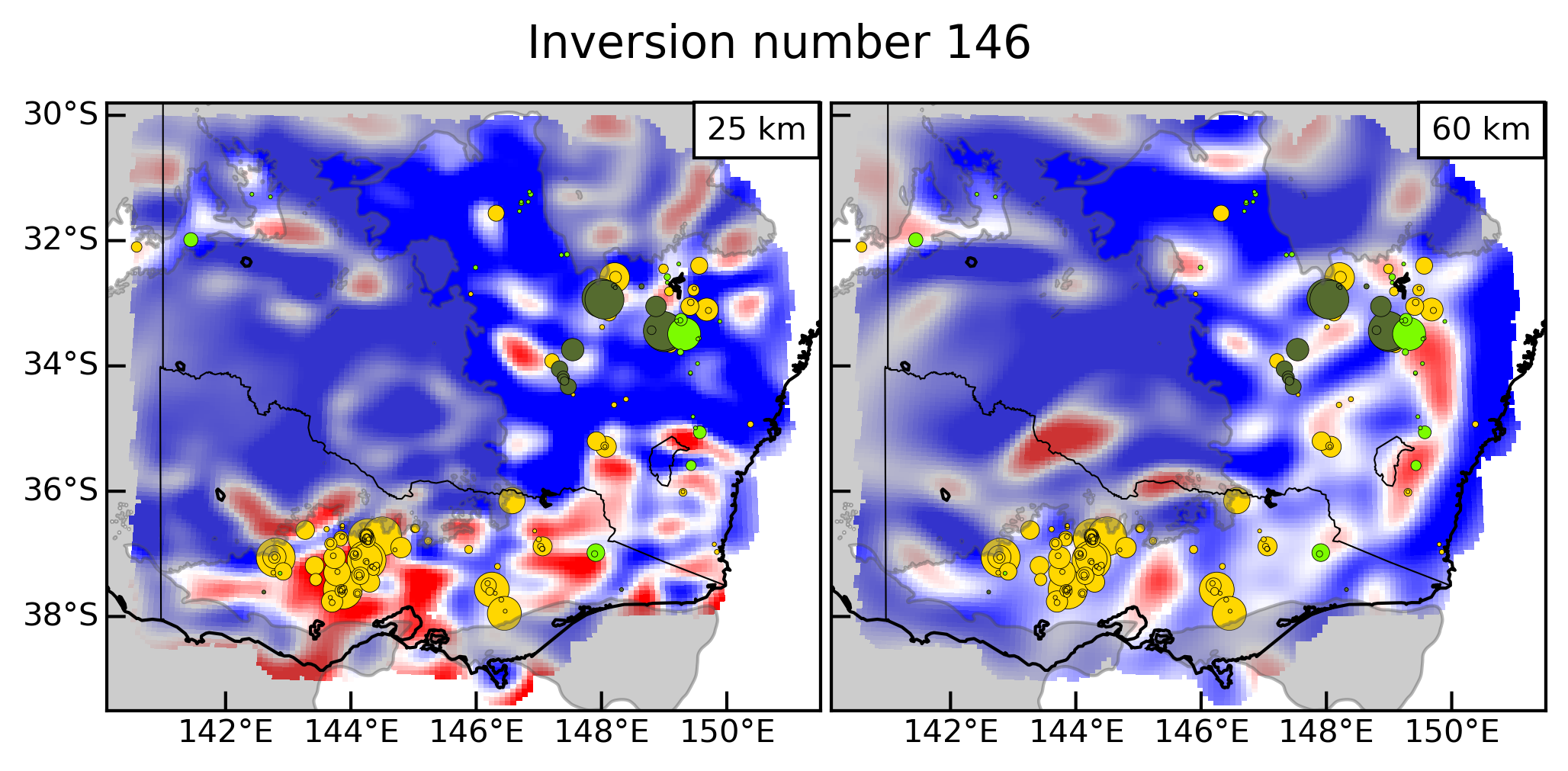

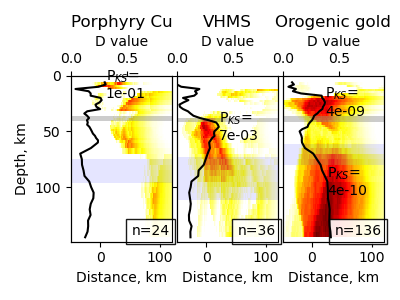


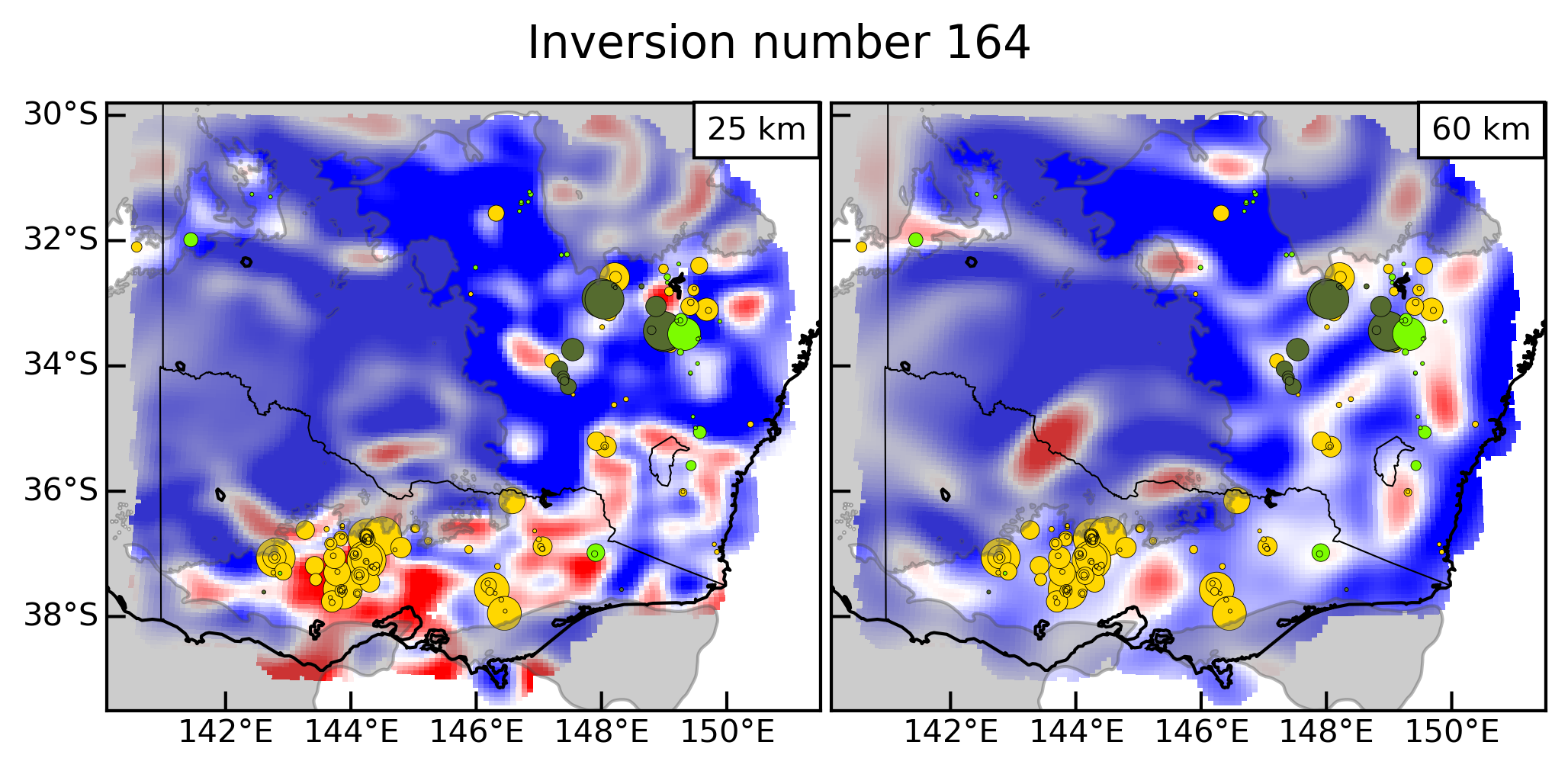

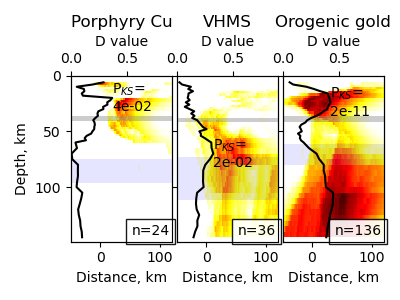


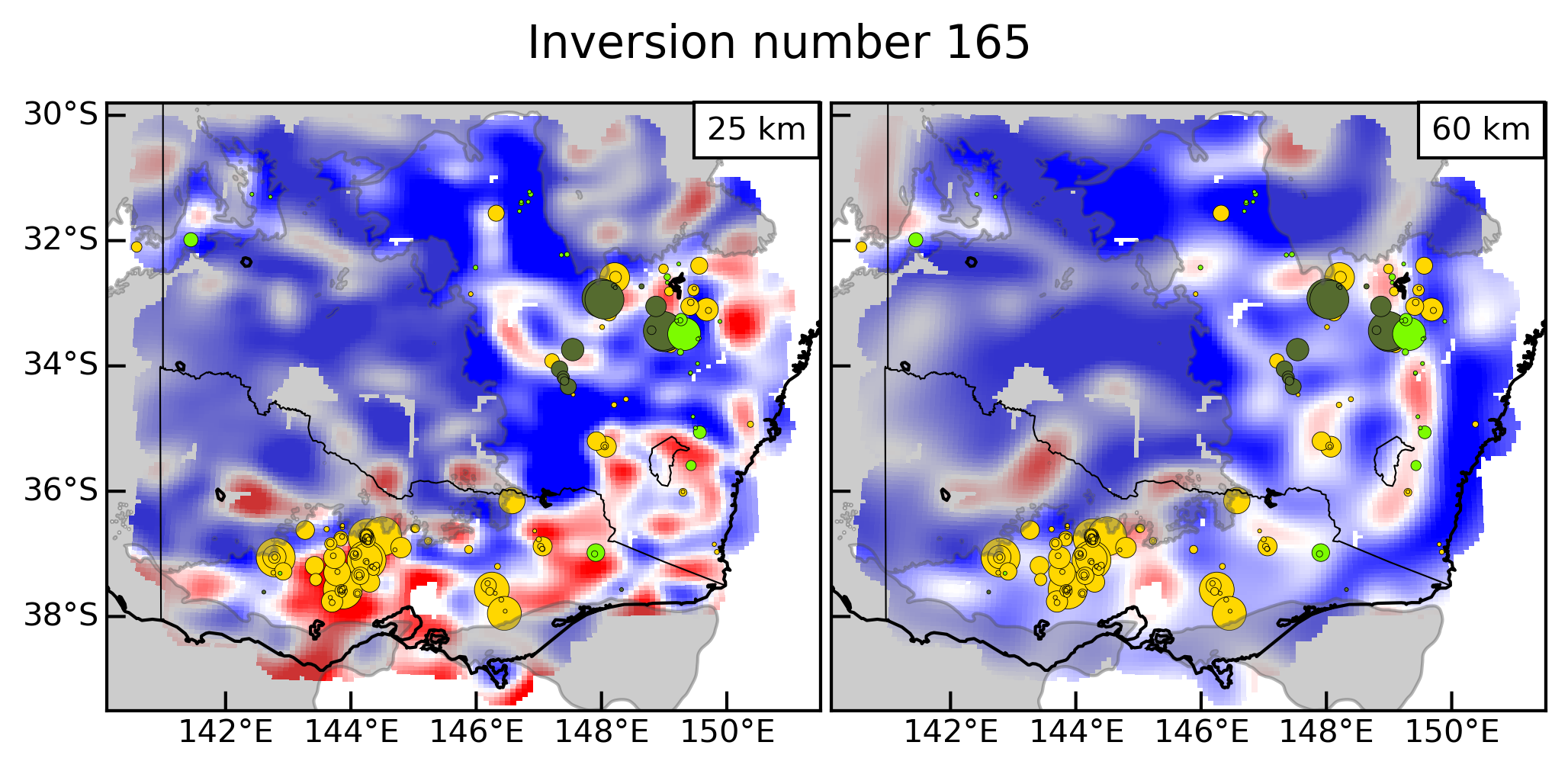

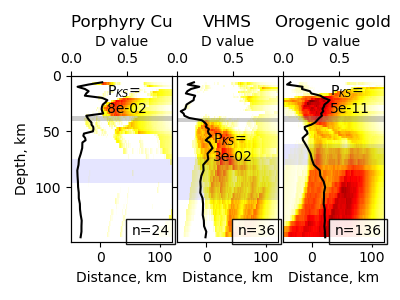


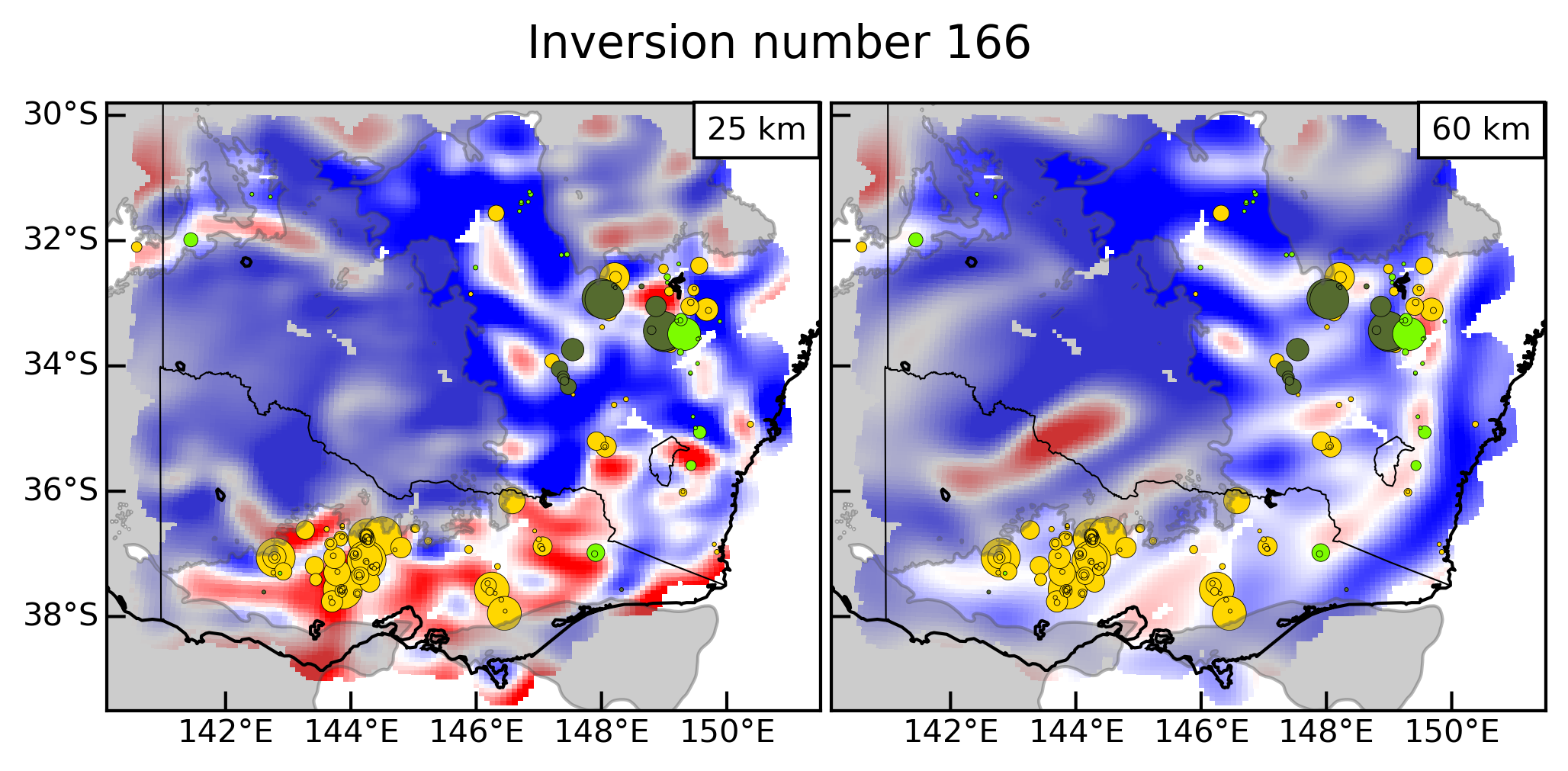

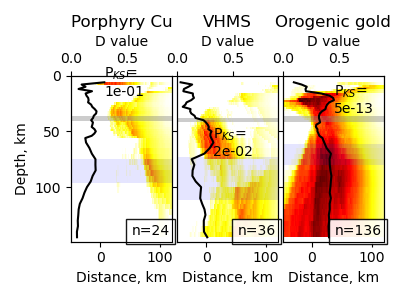


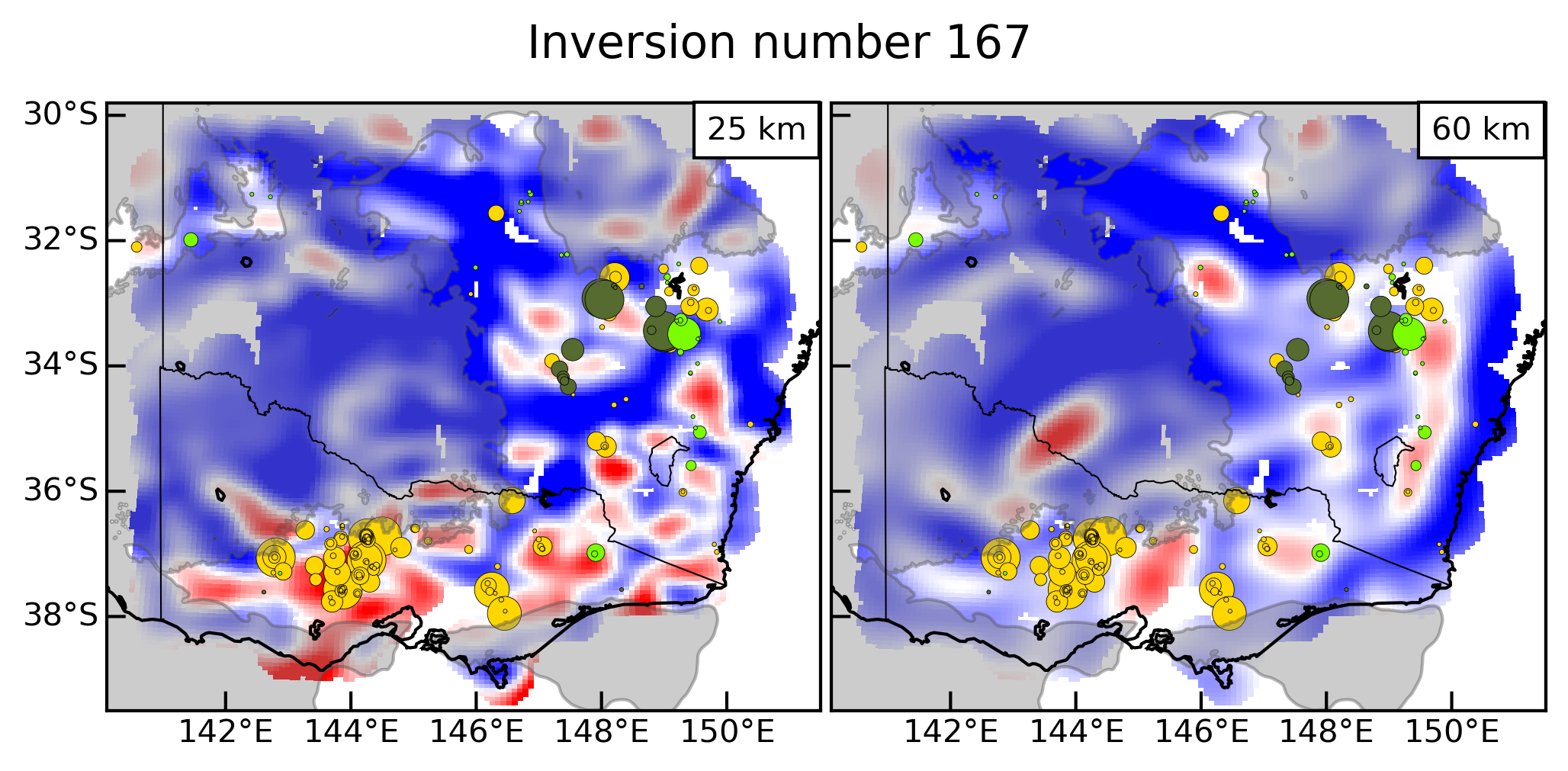

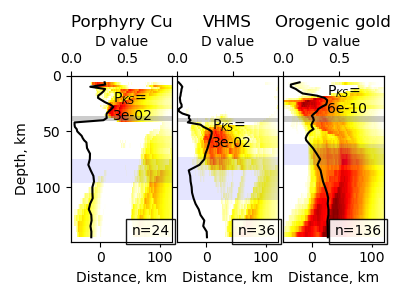


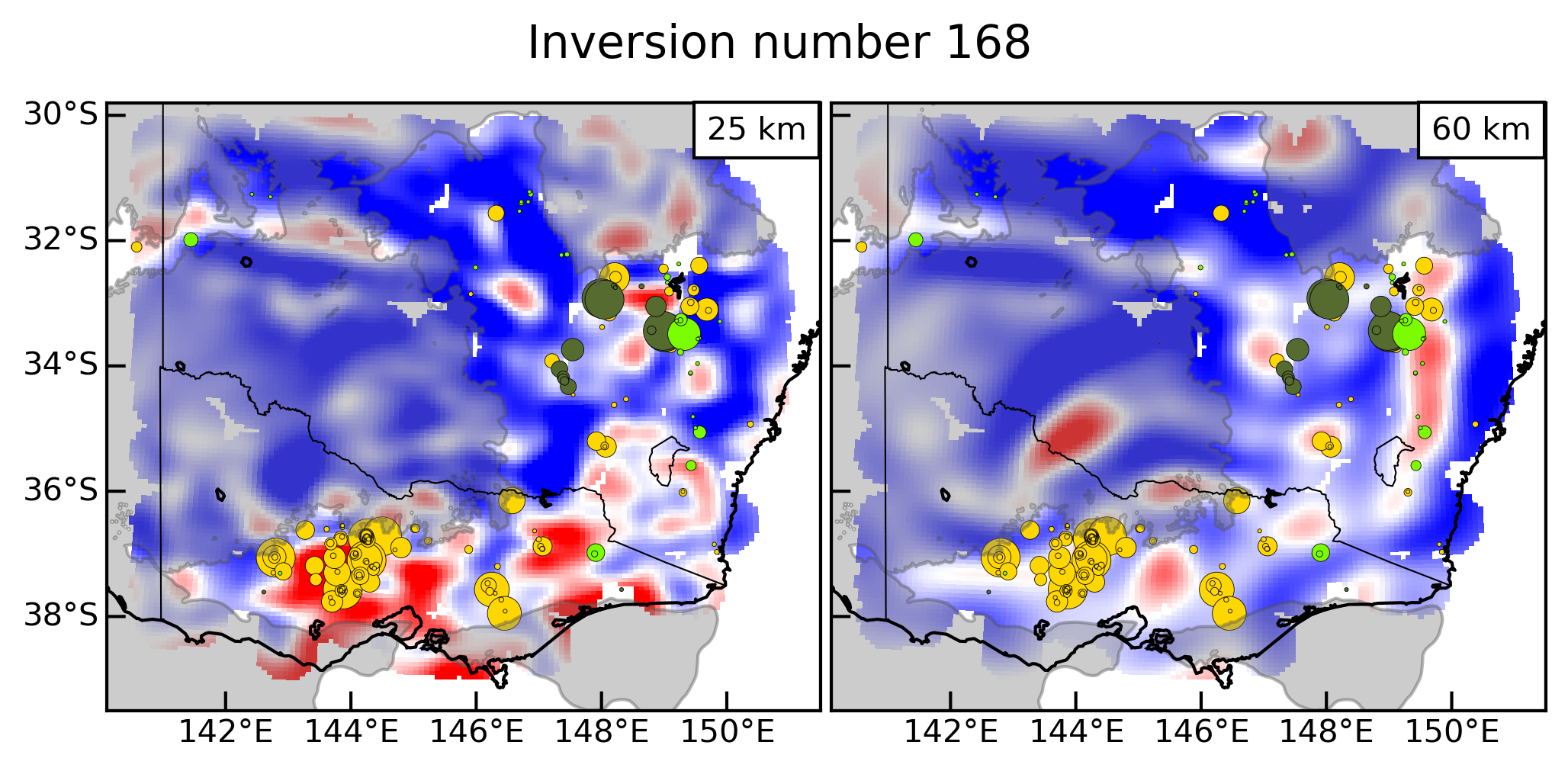

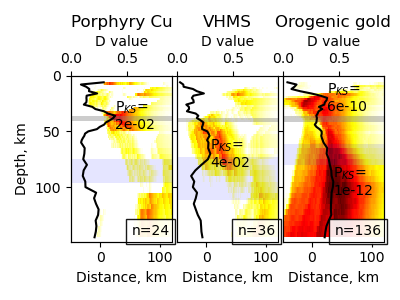


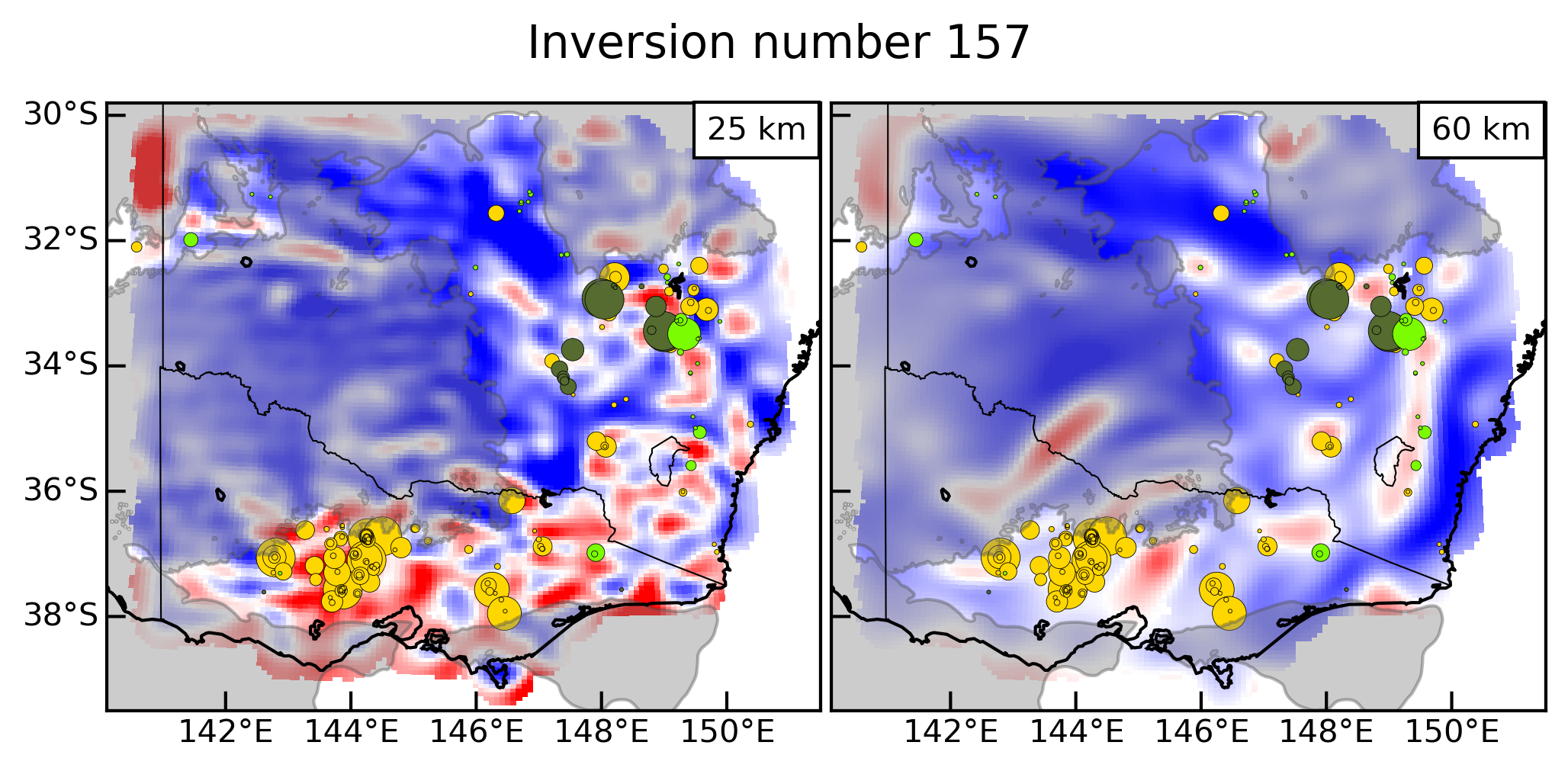

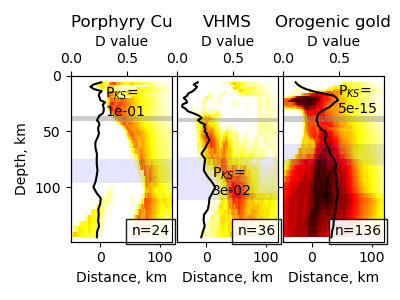


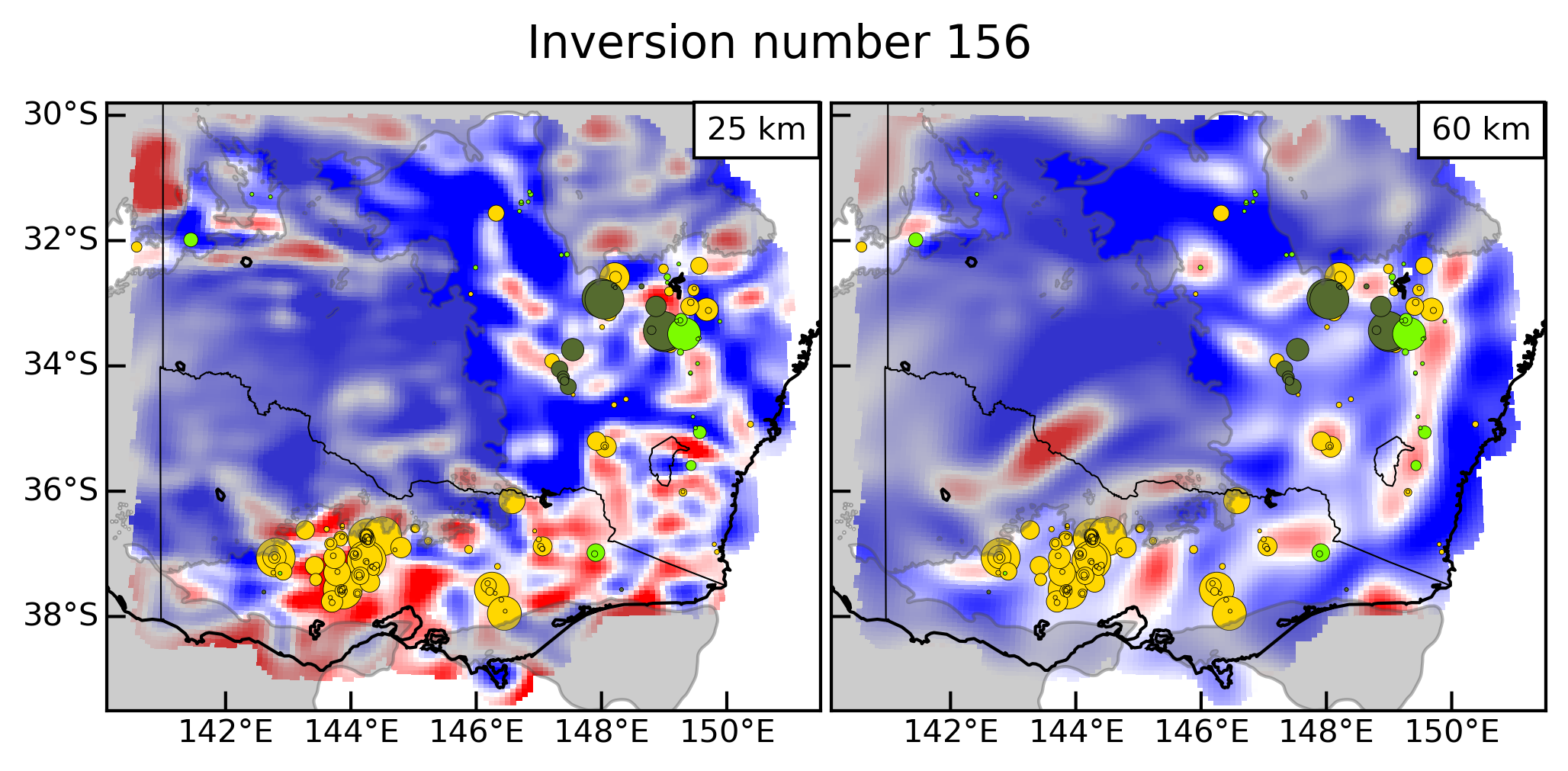

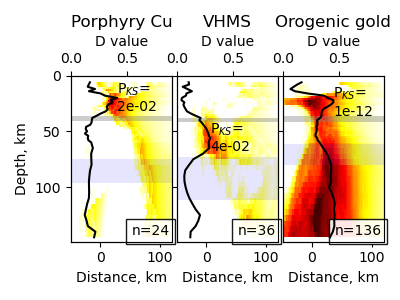


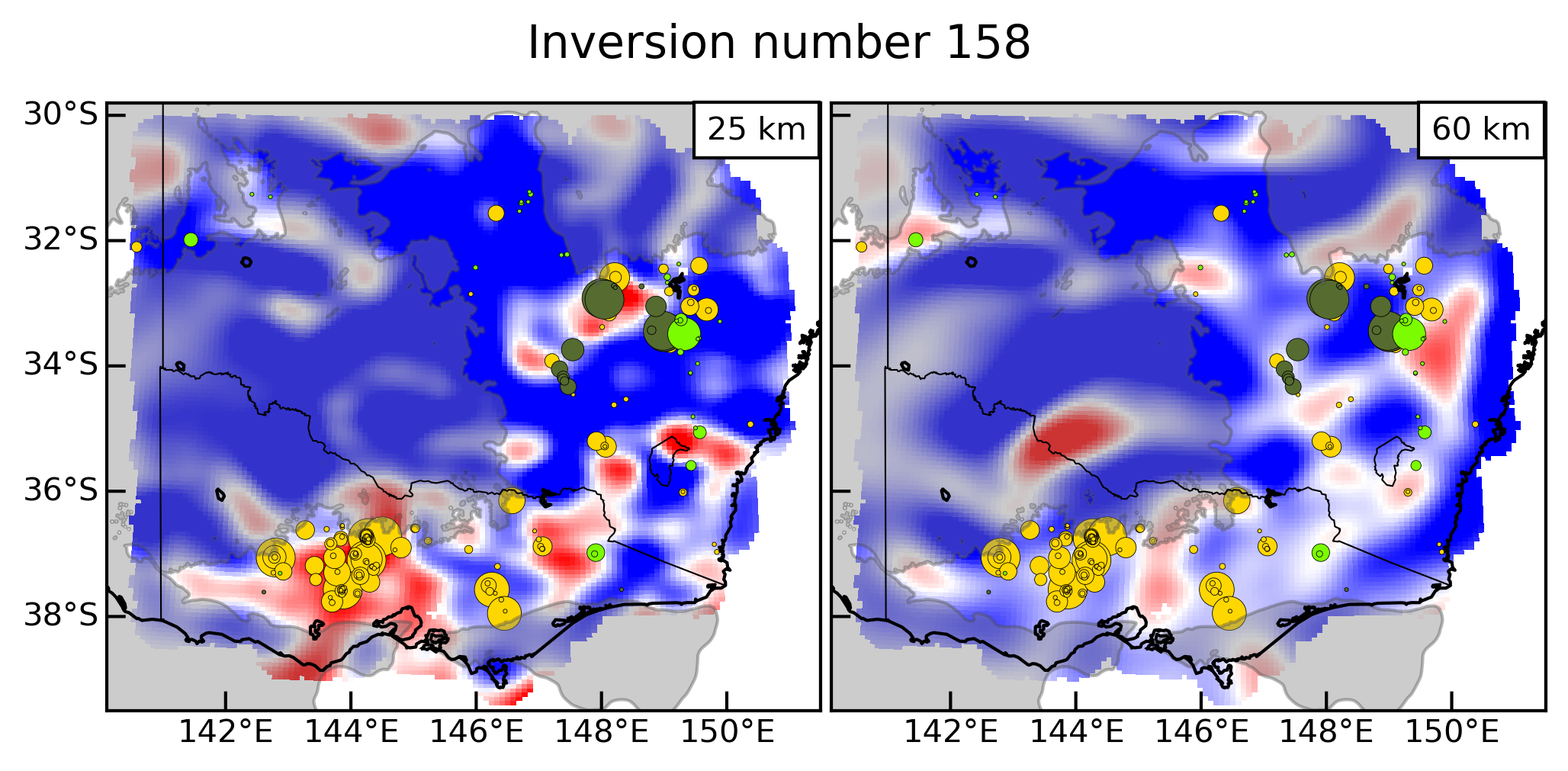

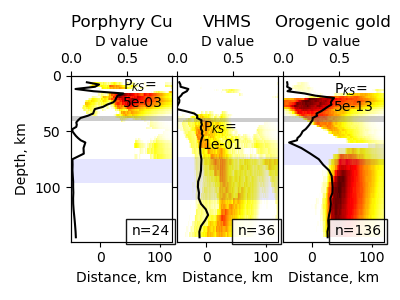


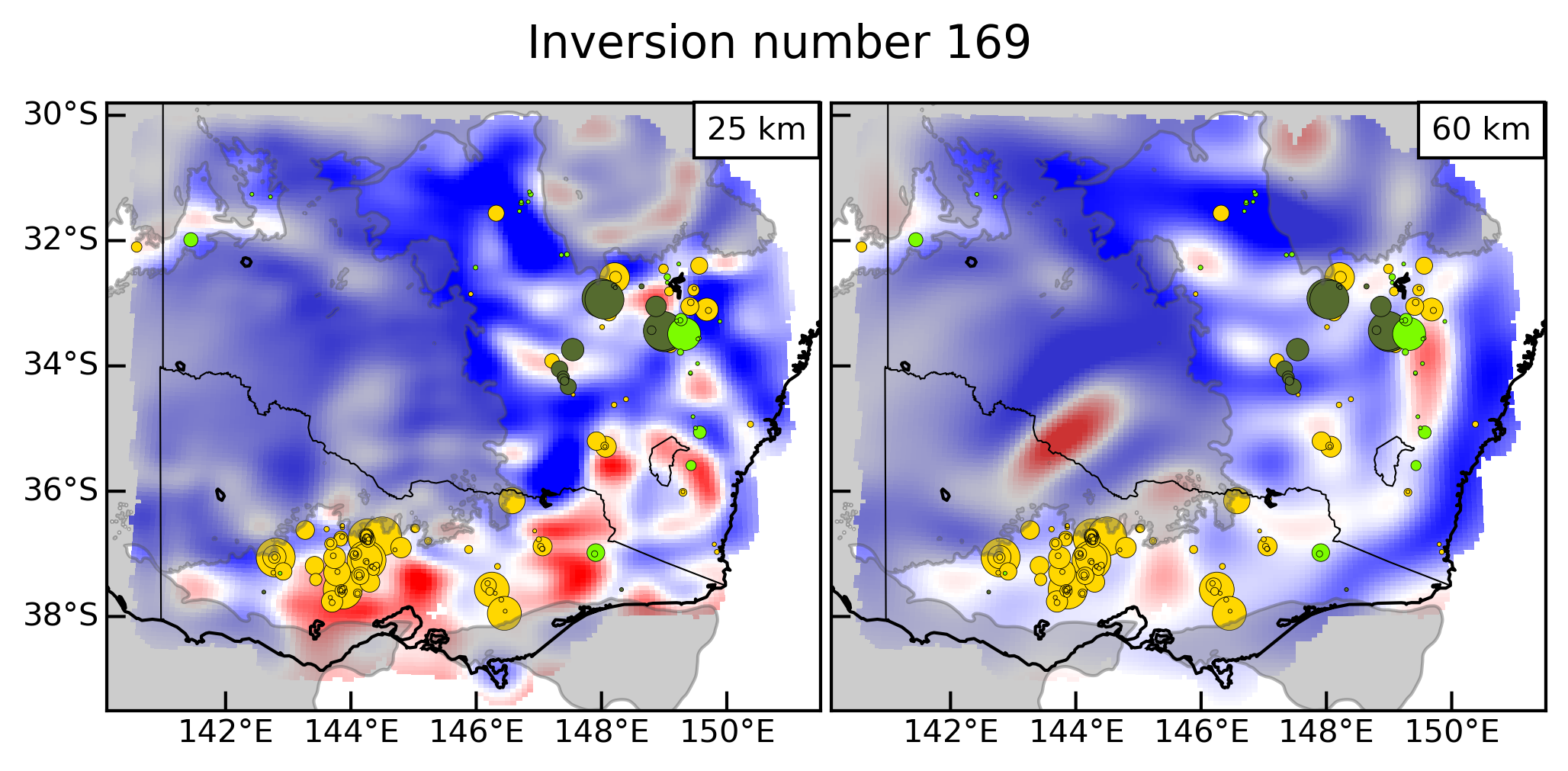

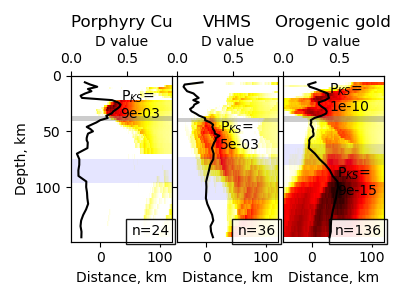


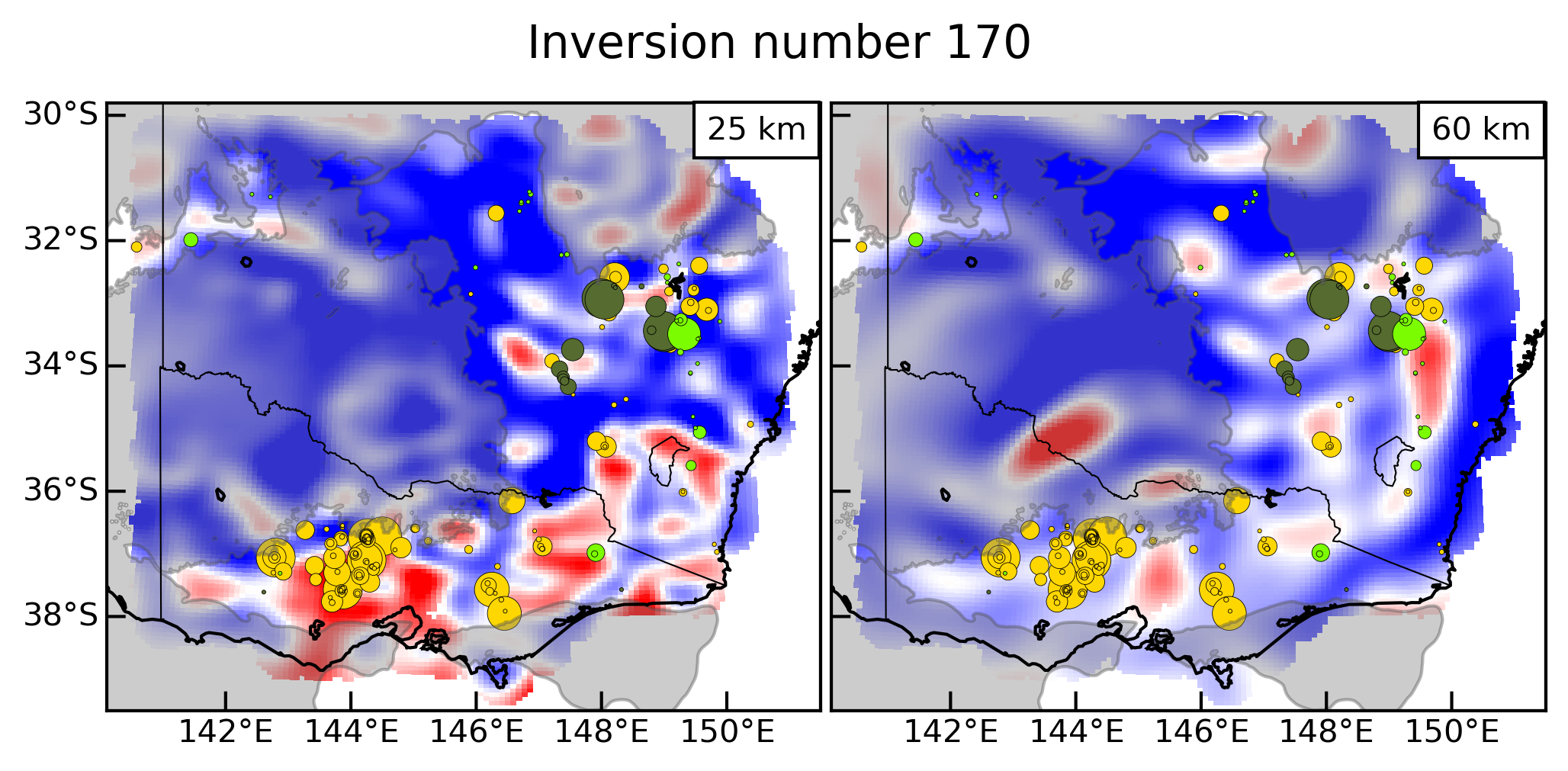

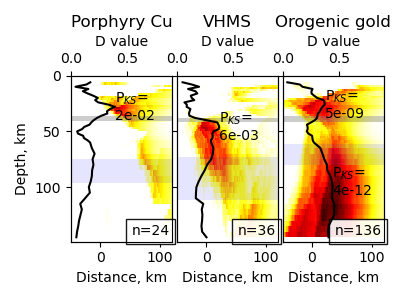


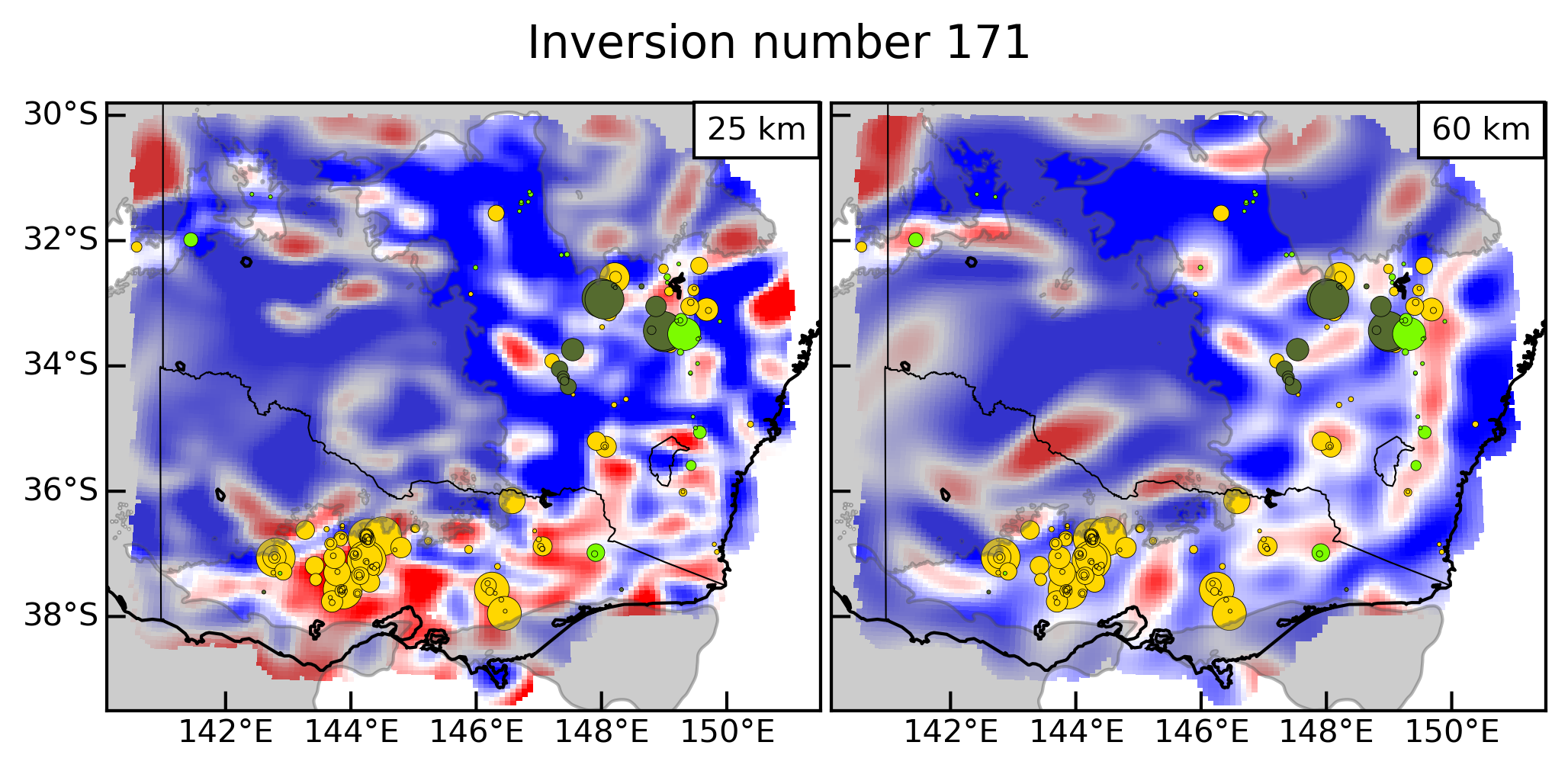

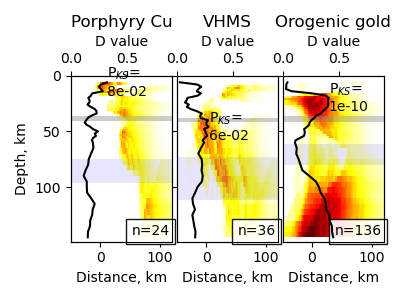


Figure 2: Summary of results of analysis for sensitivity tests listed in Table 1. Key inversion parameters for each sensitivity test are described in Table 1. Left two panels: Depth slices at 25 km and 60 km, with gold deposit locations. Deposits are colored by deposit style and sized by total contained resource, with symbol color and size as for Figure 1 of main manuscript. Mesozoic to Cenozoic sedimentary basin cover^1^ shown semi-transparent in grey. Right panel: Difference, D, between the cumulative distribution function of the distance from the 100 $\Omega$.m contour from deposits (CDF${}_{deposits}$) compared to random locations (CDF${}_{random}$), for porphyry copper, volcanic-hosted massive sulfide, and orogenic gold, in southeast Australia, for each sensitivity test.

1. Analysis using different deposit size thresholds

In this section we show the results of statistical analysis with different thresholds applied on contained resource for deposits analyzed. Three thresholds were analyzed: deposits with either ≥0.1 t Au or ≥100 t Cu, deposits with either ≥1 t Au or ≥1000t Cu, and deposits with either ≥10 t Au or ≥10000 t Cu.

There is little difference with deposit size in terms of the cumulative distribution functions as a function of depth, i.e. orogenic gold shows peaks in the mid-lower crust and asthenospheric mantle, VHMS shows a peak in the lithospheric mantle, and porphyry copper shows a subtle peak in the mid to lower crust. However, as the threshold increases, there is an increase in the minimum Kolmogorov-Smirnov parameter for all deposit styles, likely due to the decreasing numbers of deposits.


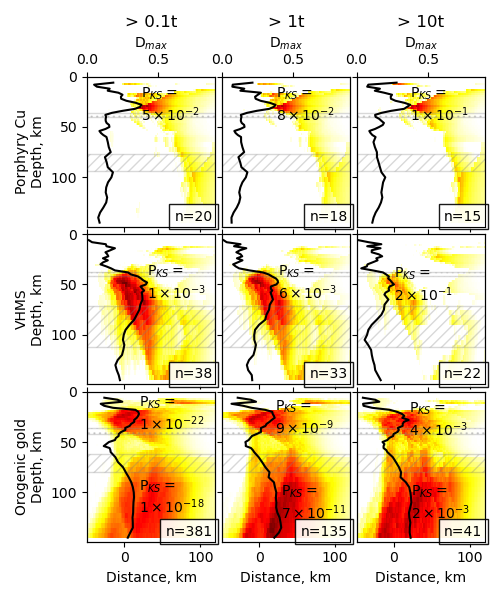


Figure 3. Difference, D, between the cumulative distribution function of the distance from the 100 $\Omega$m contour from deposits (CDF${}_{deposits}$) compared to random locations (CDF${}_{random}$), for porphyry copper, volcanic-hosted massive sulfide, orogenic gold and intrusion-related gold, in southeast Australia. The left panel shows results including deposits with either >0.1 t Au or >100 t Cu, the middle panel shows results including deposits with either >1 t Au or >1,000 t Cu, the right panel shows results including deposits with either >10 t Au or >10 Mt Cu.

1. Different resistivity contours

Here we show statistical analysis of distance to different resistivity contours using the southeast Australian resistivity model: 100 Ω.m (as in main paper), as well as 50 Ω.m (i.e. half of this value) and 200 Ω.m (double). These values encompass the reference models used for all the inversions presented in the main paper. The plots show that for porphyry copper and VHMS, there are little difference in terms of the cumulative distribution as a function of depth for different resistivity contours. For orogenic gold, the mantle peak is less defined when the 50 Ω.m contour is used which might reflect that the anomalies are generally more diffuse and less conductive in this region of the resistivity models.


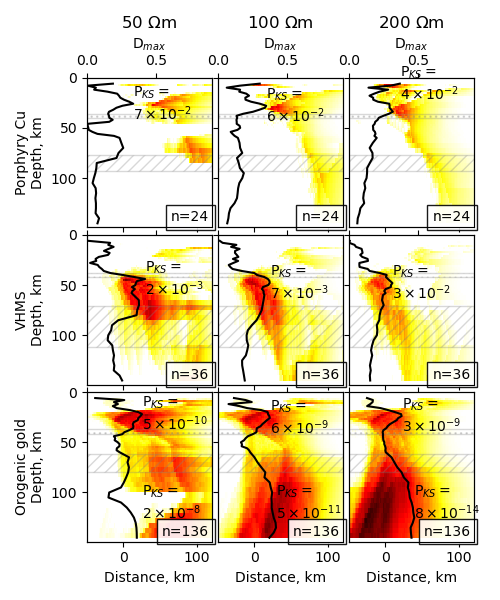


Figure 4: Difference, D, between the cumulative distribution function of the distance from the 50 Ω.m contour (top panel), the 100 Ω.m contour (middle panel) and the 200 Ω.m contour from deposits (CDF_deposits_) compared to random locations (CDF_random_), for porphyry copper, volcanic-hosted massive sulfide, orogenic gold and intrusion-related gold, in southeast Australia.

1. Resistivity modelling – temperature and water content

In order to examine whether the anomalies in southeast Australia that are associated with mineral deposits can be explained by temperature and hydration status alone, or if additional phases are required, we calculate resistivity as a function of temperature and water content at several depths in the mantle using relationships developed from laboratory measurements on dry and hydrated mantle minerals^2-5^. The mantle was assumed to have a composition of 65% olivine, 8% clinopyroxene, 17% orthopyroxene and 10% garnet^6^. The calculation for hydrated mineralogies were taken using maximum reasonable contents^7^ with dry formulation used for garnet in both cases as the maximum water that can be contained in garnet is ≤ 3 ppm^7^. Calculations were carried out using two temperature models^8,9^. Geometric mean and arithmetic mean of conductivity (parallel model, in which the conductive phases are assumed to be fully connected) were used to calculate overall composition. These means can be taken to represent end-member mixing models, with a geometric mean likely to provide a better estimate if the more conductive pyroxene minerals are randomly distributed throughout the mantle, tending toward a parallel model if these minerals are concentrated in metasomatic veins as inferred beneath the Buckland Volcanic Province in Queensland^10^.

These calculations show that at 40-50 km depth, hydrated mineralogies are likely required to explain the resistivities across most of the model area. Furthermore, most of the conductors that are spatially associated with mineral deposits require additional mineral phases other than hydrated mantle minerals, to explain the resistivity anomalies. At 60 km depth, hydrous phases are required across much of the model space, and additional phases are needed in the northwest of the model to explain the inverted resistivities. At 100 km, the resistivity anomalies can largely be explained by hydration status alone, and in many places dry mineralogy can explain the resistivities in the model.


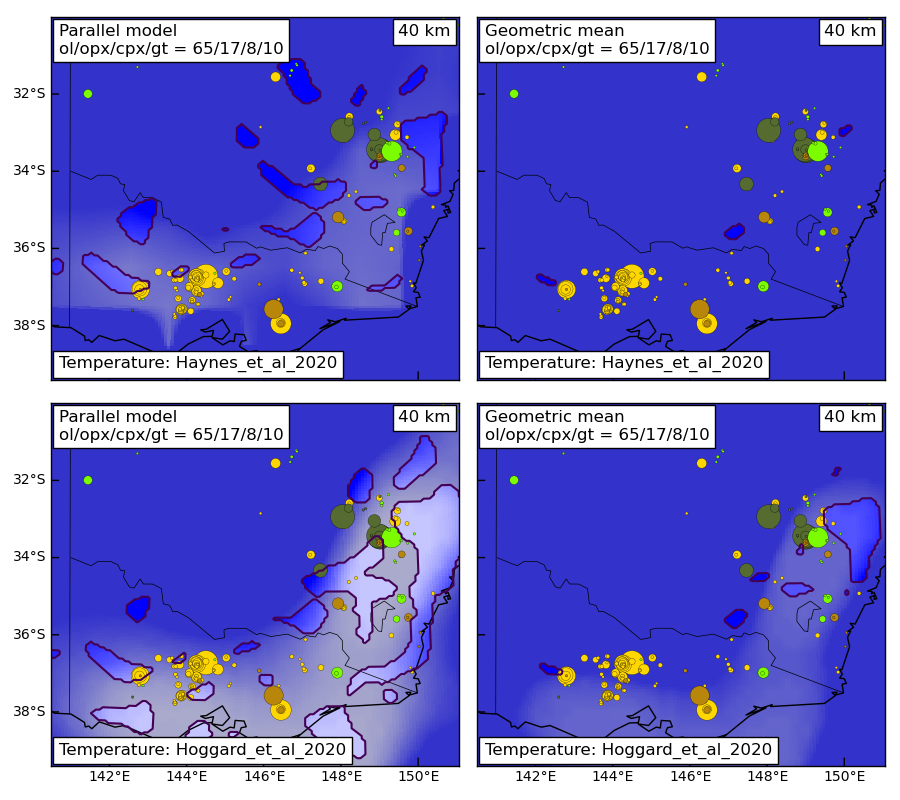


Figure 5: Calculated resistivity at 40 km depth using formulation for dry mantle minerals as a function of temperature based on laboratory measurements from clinopyroxne (cpx; Yang et al., 2011), orthopyroxene (opx; Yang et al., 2012), olivine (ol; Wang et al., 2006) and garnet (gnt; Jones et al., 2012). Temperature models from Hoggard et al. (2020) (bottom) and Haynes et al. (2020) (top) and mantle composition from Griffin et al. (2009). Overall resistivity was calculated using the arithmetic mean of conductivity (parallel model) and geometric mean. Grey shaded areas show where inverted resistivity is lower than calculated resistivity and hence additional conductive phases would be required to get the inverted resistivities. Deposit locations and resistivity color scale shown as in Figure 1 of main manuscript.


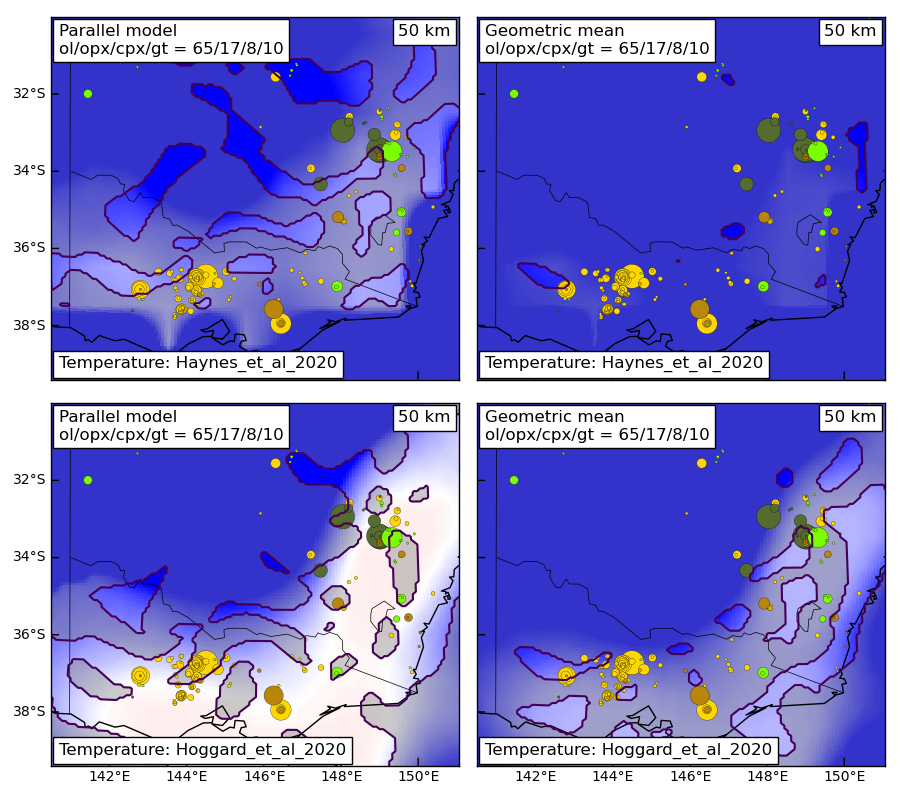


Figure 6. Calculated resistivity at 50 km depth using formulation for dry mantle minerals as a function of temperature based on laboratory measurements from clinopyroxne (cpx; Yang et al., 2011), orthopyroxene (opx; Yang et al., 2012), olivine (ol; Wang et al., 2006) and garnet (gnt; Jones et al., 2012). Temperature models from Hoggard et al. (2020) (bottom) and Haynes et al. (2020) (top) and mantle composition from Griffin et al. (2009). Overall resistivity was calculated using the arithmetic mean of conductivity (parallel model) and geometric mean. Grey shaded areas show where inverted resistivity is lower than calculated resistivity and hence additional conductive phases would be required to get the inverted resistivities. Deposit locations and resistivity color scale shown as in Figure 1 of main manuscript.


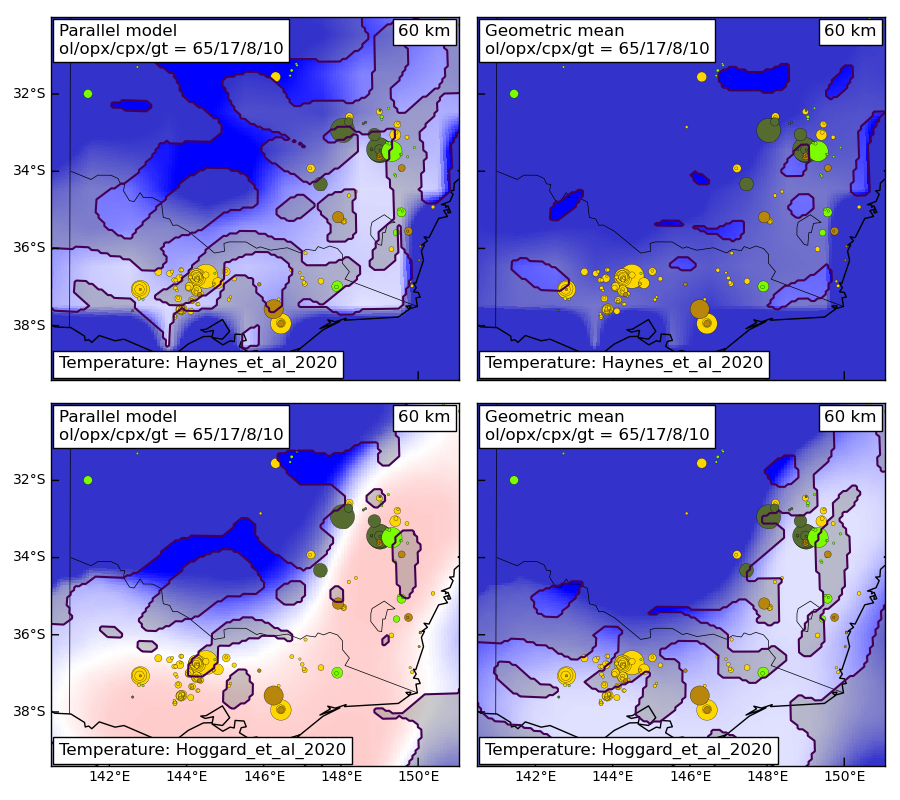


Figure 7. Calculated resistivity at 60 km depth using formulation for dry mantle minerals as a function of temperature based on laboratory measurements from clinopyroxne (cpx; Yang et al., 2011), orthopyroxene (opx; Yang et al., 2012), olivine (ol; Wang et al., 2006) and garnet (gnt; Jones et al., 2012). Temperature models from Hoggard et al. (2020) (bottom) and Haynes et al. (2020) (top) and mantle composition from Griffin et al. (2009). Overall resistivity was calculated using the arithmetic mean of conductivity (parallel model) and geometric mean. Grey shaded areas show where inverted resistivity is lower than calculated resistivity and hence additional conductive phases would be required to get the inverted resistivities. Deposit locations and resistivity color scale shown as in Figure 1 of main manuscript.


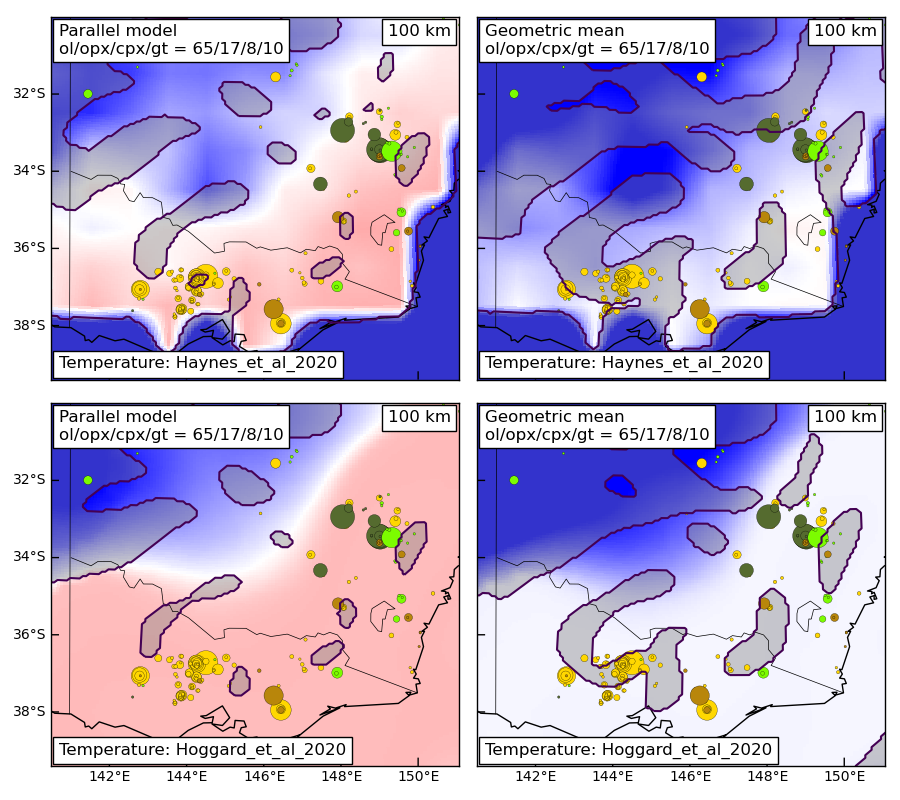


Figure 8: Calculated resistivity at 100 km depth using formulation for dry mantle minerals as a function of temperature based on laboratory measurements from clinopyroxne (cpx; Yang et al., 2011), orthopyroxene (opx; Yang et al., 2012), olivine (ol; Wang et al., 2006) and garnet (gnt; Jones et al., 2012). Temperature models from Hoggard et al. (2020) (bottom) and Haynes et al. (2020) (top) and mantle composition from Griffin et al. (2009). Overall resistivity was calculated using the arithmetic mean of conductivity (parallel model) and geometric mean. Grey shaded areas show where inverted resistivity is lower than calculated resistivity and hence additional conductive phases would be required to get the inverted resistivities. Deposit locations and resistivity color scale shown as in Figure 1 of main manuscript.


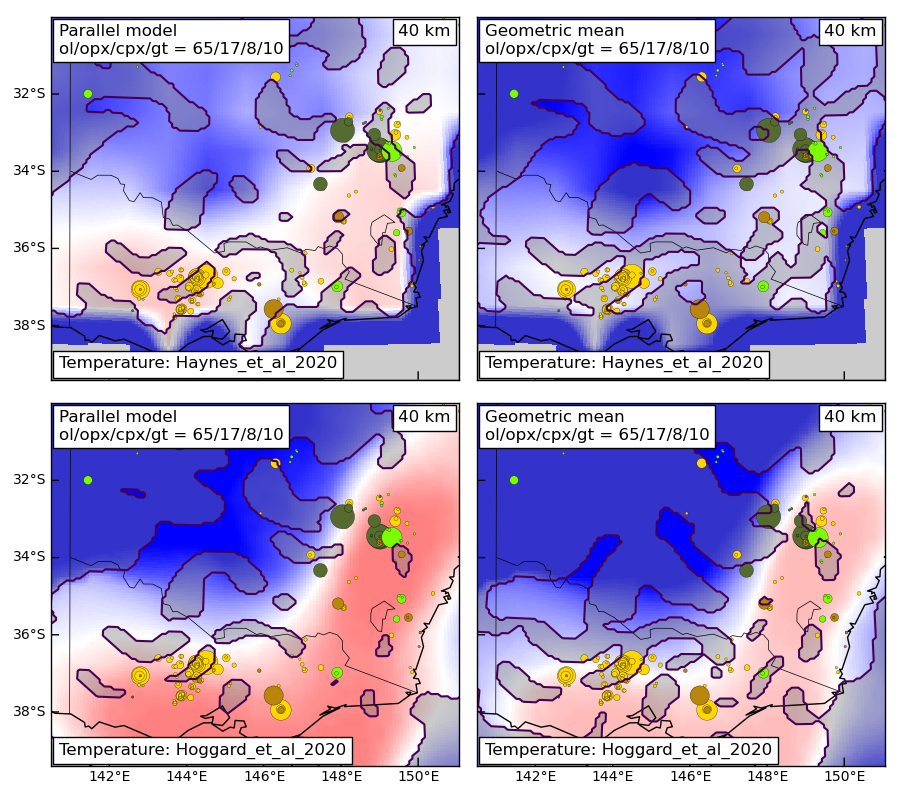


Figure 9. Calculated resistivity at 40 km depth using formulation for wet mantle minerals as a function of temperature based on laboratory measurements from clinopyroxne (cpx; Yang et al., 2011), orthopyroxene (opx; Yang et al., 2012), olivine (ol; Wang et al., 2006) and garnet (gnt; Jones et al., 2012). Temperature models from Hoggard et al. (2020) (bottom) and Haynes et al. (2020) (top) and mantle composition from Griffin et al. (2009). Overall resistivity was calculated using the arithmetic mean of conductivity (parallel model) and geometric mean. Grey shaded areas show where inverted resistivity is lower than calculated resistivity and hence additional conductive phases would be required to get the inverted resistivities. Deposit locations and resistivity color scale shown as in Figure 1 of main manuscript.


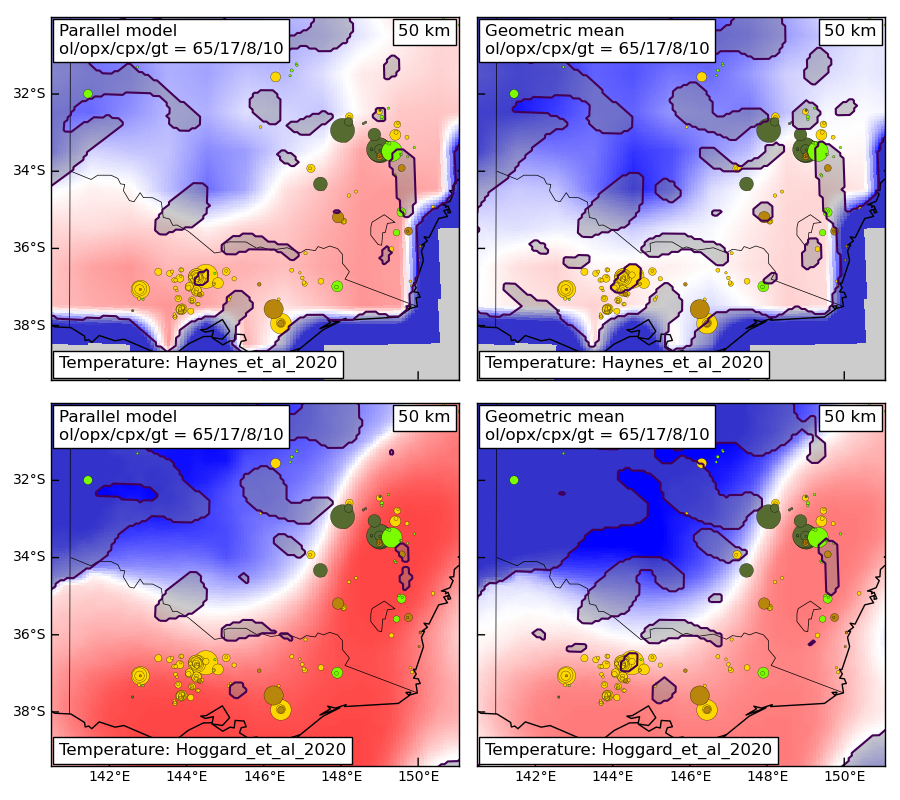


Figure 10. Calculated resistivity at 50 km depth using formulation for wet mantle minerals as a function of temperature based on laboratory measurements from clinopyroxne (cpx; Yang et al., 2011), orthopyroxene (opx; Yang et al., 2012), olivine (ol; Wang et al., 2006) and garnet (gnt; Jones et al., 2012). Temperature models from Hoggard et al. (2020) (bottom) and Haynes et al. (2020) (top) and mantle composition from Griffin et al. (2009). Overall resistivity was calculated using the arithmetic mean of conductivity (parallel model) and geometric mean. Grey shaded areas show where inverted resistivity is lower than calculated resistivity and hence additional conductive phases would be required to get the inverted resistivities. Deposit locations and resistivity color scale shown as in Figure 1 of main manuscript.


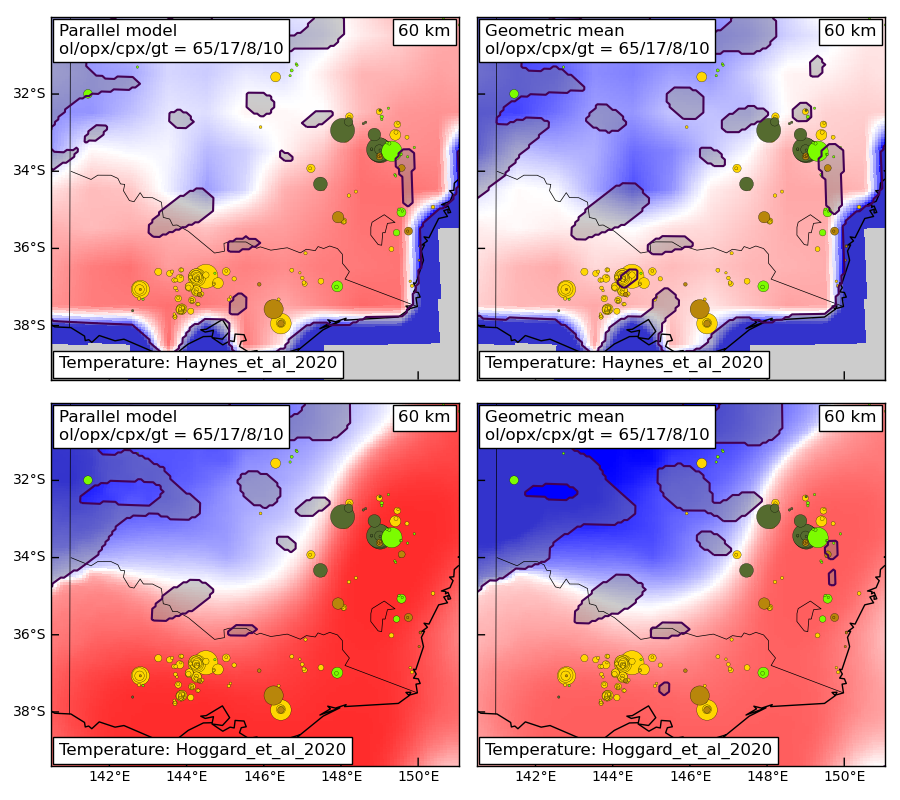


Figure 11. Calculated resistivity at 60 km depth using formulation for wet mantle minerals as a function of temperature based on laboratory measurements from clinopyroxne (cpx; Yang et al., 2011), orthopyroxene (opx; Yang et al., 2012), olivine (ol; Wang et al., 2006) and garnet (gnt; Jones et al., 2012). Temperature models from Hoggard et al. (2020) (bottom) and Haynes et al. (2020) (top) and mantle composition from Griffin et al. (2009). Overall resistivity was calculated using the arithmetic mean of conductivity (parallel model) and geometric mean. Grey shaded areas show where inverted resistivity is lower than calculated resistivity and hence additional conductive phases would be required to get the inverted resistivities. Deposit locations and resistivity color scale shown as in Figure 1 of main manuscript.


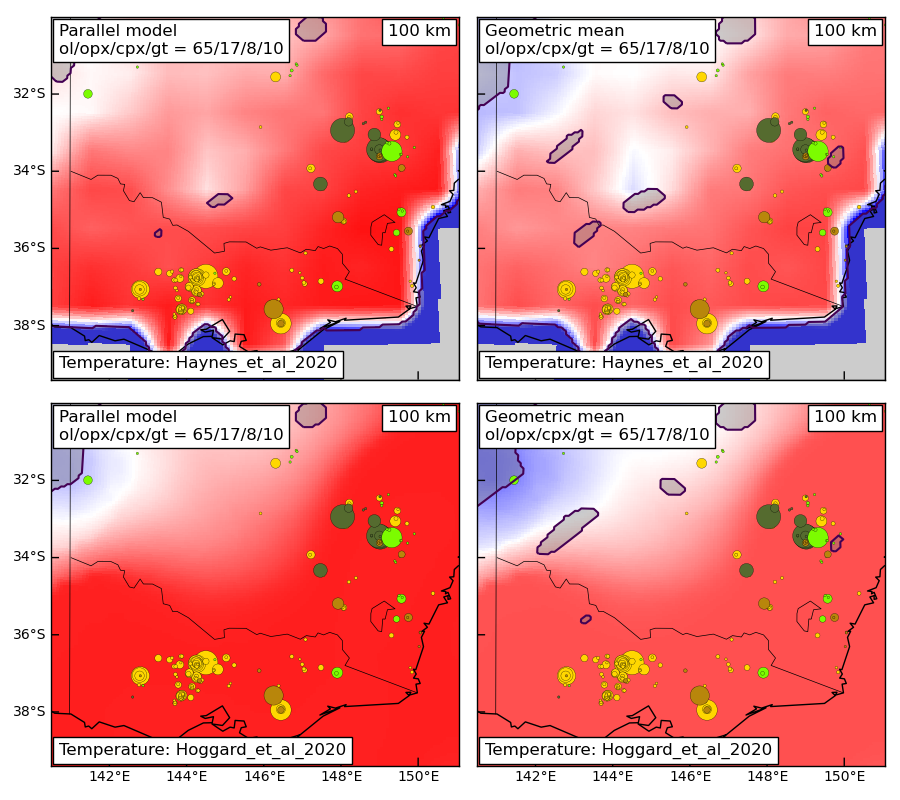


Figure 12. Calculated resistivity at 100 km depth using formulation for wet mantle minerals as a function of temperature based on laboratory measurements from clinopyroxne (cpx; Yang et al., 2011), orthopyroxene (opx; Yang et al., 2012), olivine (ol; Wang et al., 2006) and garnet (gnt; Jones et al., 2012). Temperature models from Hoggard et al. (2020) (bottom) and Haynes et al. (2020) (top) and mantle composition from Griffin et al. (2009). Overall resistivity was calculated using the arithmetic mean of conductivity (parallel model) and geometric mean. Grey shaded areas show where inverted resistivity is lower than calculated resistivity and hence additional conductive phases would be required to get the inverted resistivities. Deposit locations and resistivity color scale shown as in Figure 1 of main manuscript.

References

1 Raymond, O. Australian Geological Provinces 2018.01 edition. Geoscience Australia, Canberra. h<ttps://ecat.ga.gov.au/geonetwork/srv/eng/catalog.search#/metadata/116823.> (2018).

2 Yang, X., Keppler, H., McCammon, C. & Ni, H. Electrical conductivity of orthopyroxene and plagioclase in the lower crust. *Contributions to Mineralogy and Petrology* **163**, 33-48, doi:10.1007/s00410-011-0657-9 (2012).

3 Yang, X. *et al.* Effect of water on the electrical conductivity of lower crustal clinopyroxene. *Journal of Geophysical Research* **116**, B04208-B04208, doi:10.1029/2010JB008010 (2011).

4 Wang, D., Mookherjee, M., Xu, Y. & Karato, S.-i. The effect of water on the electrical conductivity of olivine. *Nature* **443**, 977-980, doi:10.1038/nature05256 (2006).

5 Jones, A. G., Evans, R. L. & Eaton, D. W. Velocity–conductivity relationships for mantle mineral assemblages in Archean cratonic lithosphere based on a review of laboratory data and Hashin–Shtrikman extremal bounds. *Lithos* **109**, 131-143, doi:<https://doi.org/10.1016/j.lithos.2008.10.014> (2009).

6 Griffin, W. L., O’Reilly, S. Y., Afonso, J. C. & Begg, G. C. The Composition and Evolution of Lithospheric Mantle: a Re-evaluation and its Tectonic Implications. *Journal of Petrology* **50**, 1185-1204, doi:10.1093/petrology/egn033 (2009).

7 Grant, K., Ingrin, J., Lorand, J. P. & Dumas, P. Water partitioning between mantle minerals from peridotite xenoliths. *Contributions to Mineralogy and Petrology* **154**, 15-34, doi:10.1007/s00410-006-0177-1 (2007).

8 Hoggard, M. J. *et al.* Global distribution of sediment-hosted metals controlled by craton edge stability. *Nature Geoscience* **13**, 504-510, doi:10.1038/s41561-020-0593-2 (2020).

9 Haynes, M. W., Fomin, I., Afonso, J. C., Gorbatov, A. & Salajegheh, F. in *Extended abstract, Exploring for the Future* 1-4 (Geoscience Australia, Canberra, 2020).

10 Shea, J. J. & Foley, S. F. Evidence for a Carbonatite-Influenced Source Assemblage for Intraplate Basalts from the Buckland Volcanic Province, Queensland, Australia. *Minerals* **9**, doi:10.3390/min9090546 (2019).
